# Mesothelin biology and the evolving landscape of targeted immunotherapy

**DOI:** 10.1016/j.omton.2026.201148

**Published:** 2026-02-07

**Authors:** Remy Boisgard, Patrick Chames, Brigitte Kerfelec

**Affiliations:** 1 Aix Marseille University, CNRS, INSERM, Institut Paoli-Calmettes, CRCM, Marseille, France

**Keywords:** mesothelin, cancer, clinical trials, targeted therapy, immunotherapy

## Abstract

Due to its restricted expression in normal tissues, its frequent overexpression in various aggressive malignancies, and its involvement in several pro-tumorigenic signaling pathways, mesothelin, a glycosylphosphatidylinositol-anchored cell surface protein, has been identified as a promising tumor-associated antigen. This review provides an up-to-date comprehensive overview regarding the role of mesothelin in cancer. It encompasses the protein structural characteristics, expression profiles, glycosylation patterns, shedding mechanisms, prognostic significance, and functional roles. Subsequently, therapeutic strategies targeting mesothelin that have advanced to clinical trial stage are discussed, including vaccine-based approaches, antibody-mediated immunotherapies, and cell therapies. The remaining challenges of mesothelin targeted treatments are highlighted, along with the ongoing options aimed at overcoming these limitations.

## Graphical abstract

**Figure:**
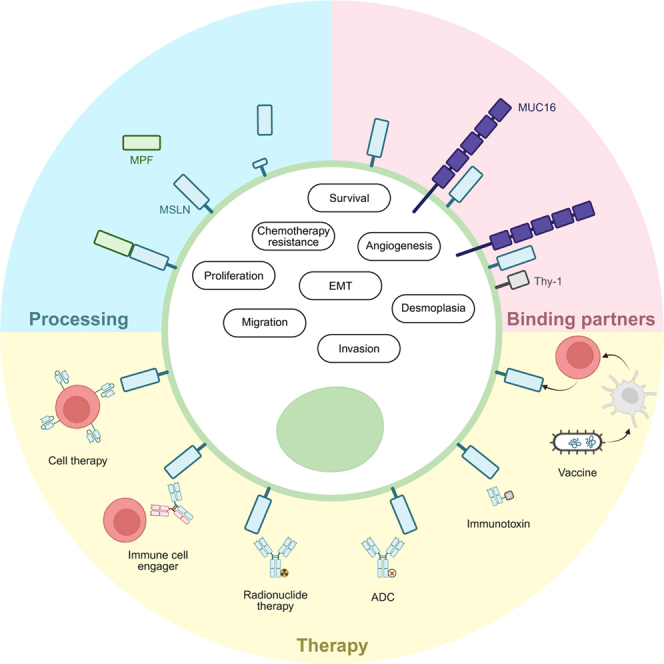

## Teaser

Tumor-restricted overexpression of mesothelin, in contrast to its limited normal tissue expression, makes it a compelling target in oncology. Boisgard and and colleagues provide an up-to-date synthesis of established and underdiscussed aspects of mesothelin biology and clinical trial details. They also underscore several questions critical for the future optimization of mesothelin-targeted therapies.

## Introduction

Discovered in 1992 by Chang and Pastan, mesothelin (MSLN) is a glycosylphosphatidylinositol (GPI)-anchored cell surface glycoprotein predominantly expressed on the mesothelial cells that line the pleural, peritoneal, and pericardial cavities.^1^^,^^2^ A recent immunohistochemical study on 76 healthy organs has revealed a broader expression of mesothelin, primarily observed in non-vital tissues in adults such as the tongue, thymus, gallbladder, fallopian tubes, uterus, and placenta.^3^

The biological role of mesothelin in healthy tissues remains to be elucidated. Its function appears non-essential, as MSLN gene invalidation experiments in mice did not result in any significant abnormalities in development, fertility, or platelet count compared with wild-type mice.^4^

It has been hypothesized that MSLN, akin to several other GPI-anchored proteins, plays a role in cell adhesion processes and signaling pathways. Sathyanarayana et al. proposed that MSLN contributes to the modeling of the extracellular matrix through its interaction with matrix glycoproteins.^5^

The only described partner of mesothelin is the glycosylated transmembrane protein mucin 16 (MUC16) (∼3–5 MDa), also known as CA125, expressed at the surface of various epithelial cells but overexpressed in several cancer types.^6^^,^^7^^,^^8^ Notably, in healthy tissues, MSLN and MUC16 are expressed at low levels and in distinct cell types, suggesting that no physiological interaction occurs under normal conditions.^3^^,^^8^

**Figure 1:**
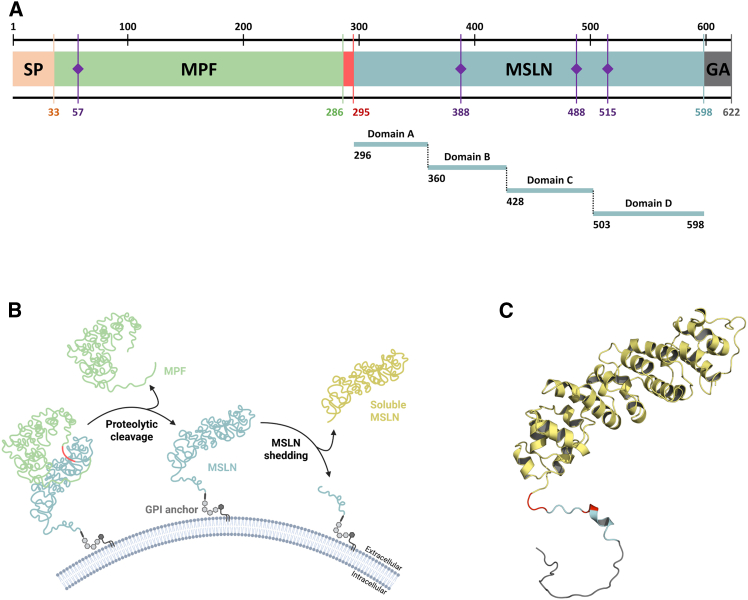
Mesothelin structure and processing (A) Mesothelin precursor is a 622-amino-acid protein with four glycosylation sites (purple) and five regions: signal peptide (SP, orange); megakaryocyte potentiating factor (MPF, green); proteolytic cleavage site (red); mature mesothelin (MSLN, blue); and the GPI anchor (GA, gray). (B) Schematic representation of the mesothelin processing from precursor to soluble mesothelin. (C) Three-dimensional AlphaFold ribbon representation of mature MSLN with major cleavage site (MCS, red) locations leading to soluble mesothelin (yellow).

The mesothelin gene is located on chromosome 16 (16p13.3) and encodes the mesothelin precursor protein (∼70 kDa).^2^ Three MSLN transcript variants have been described.^9^ Variant 1 is regarded as the canonical form because it is the predominant form expressed in both healthy and tumor tissues (Figure 1A).^10^

The MSLN precursor is transported and anchored to the cell membrane through a GPI anchor (Figure 1B). Subsequently, the MSLN precursor is cleaved by proteolysis, releasing the N-terminal soluble fragment, designated as megakaryocyte potentiating factor (MPF) (∼30 kDa). This process leaves the mature form of MSLN (∼40 kDa) anchored to the membrane.^11^ A furin cleavage site (RPRFRR) located between amino acids 286 and 295 has been identified as the primary proteolytic processing site. However, several recent studies have shown that alternative proteolytic sites are also present in this region.^12^^,^^13^

The membrane-bound mature MSLN can be shed from cell surface under normal conditions, contributing to serum MSLN levels (Figure 1C).^14^^,^^15^ However, the rate, the specific protease profile, the regulatory pathways as well as its functional significance are not documented.

The three-dimensional structure of mature MSLN has been recently determined in complex with therapeutic antibodies.^16^^,^^17^ In accordance with the predicted structure, MSLN exhibits a curved super-helical structure comprising a series of ARM (three helices) and HEAT (two helices) repeats.^5^ MSLN is organized in four domains: A (296–359), B (360–427), C (428–502), and D (503–598) (Figure 1A).^16^

## Mesothelin in cancer

### Expression

Mesothelin overexpression has been demonstrated in a multitude of tumor types, as evidenced by an extensive immunohistochemical analysis of 15,050 tumors of 122 different types. The analysis revealed that cancers with the highest prevalence and expression levels of MSLN include all types of ovarian cancer, endometrial carcinoma, pancreatic adenocarcinoma, malignant mesothelioma of the epithelioid type, and adenocarcinoma of the lung, stomach, esophagus, and colorectum.^3^ Further immunohistochemical analysis revealed a predominant membranous localization, with a less frequent cytoplasmic localization, which is likely due to the detection of the MSLN precursor not yet anchored to the membrane.^18^^,^^19^ Weidemann’s study corroborates previous results obtained in a transcriptomic study involving 19,746 samples covering 41 tumor types.^20^ Recently, a transcriptomic study of 2051 pediatric and young adult patients with acute myeloid leukemia (AML) revealed that MSLN overexpression is not exclusive to solid cancer.^21^ The authors demonstrated that MSLN is overexpressed in approximately 36% of AML cases, despite its absence in normal bone marrow.

The mechanisms leading to MSLN overexpression in tumors have yet to be elucidated, but several potential mechanisms have been postulated. As demonstrated in the literature, mesothelioma, lung, ovarian, and pancreatic cancers with constitutive activation of the Wnt signaling pathway exhibit high mesothelin expression.^12^ The presence of an 18-base-pair enhancer sequence, CanScript, that drives high levels of gene expression has been described in the MSLN promoter region in different MSLN-overexpressing cancer lines.^22^ However, the co-factor involved in the regulation of MSLN expression remains to be identified. The hypothesis of an epigenetic regulation has been postulated subsequent to the observation of the hypomethylation of the MSLN promoter in pancreatic tumor samples.^23^ However, this phenomenon was not observed in other tumors overexpressing MSLN, such as ovarian cancer. In the context of ovarian cancer, Park and Kim have demonstrated an upregulation of MSLN and MUC16 expression through the stimulation of TLR5 and TLR7 (Toll-like receptors). This upregulation is mediated by the activation of WAVE3, a member of the Wiskott-Aldrich syndrome protein (WASP) family. WAVE3 has been identified as a significant driver of the invasive and metastatic phenotypes in multiple cancer types.^24^

### Mesothelin binding partners

The only well-described partner of mesothelin is MUC16, overexpressed in several cancer types.^6^^,^^7^^,^^8^ MSLN/MUC16 interaction is a feature specific to malignant cells linked to the co-expression of MUC16 and MSLN.

Mesothelin-MUC16 binding is mediated by protein-protein interaction between domain A (296–359) of MSLN and MUC16 SEA (sea urchin sperm protein, enterokinase, and agrin) modules.^7^^,^^25^ Involvement of the SEA modules suggests that MUC16 might engage MSLN molecules, thereby generating an avidity phenomenon at the cell surface. However, enzymatic removal of MUC16 N-glycans disrupts MUC16/MSLN interaction, indicating that glycans, by maintaining MUC16 stability, are structurally critical for the binding.^26^

Recently, it has been shown that, on portal fibroblasts, MSLN can also bind to Thy-1 (thymocyte differentiation antigen 1, CD90) and form an MSLN/MUC16/Thy-1 complex in response to pro-fibrotic signals, such as transforming growth factor β1 (TGF-β1). This complex by modulating TGF-β1/TGF-βR1 signaling promotes fibroblast activation and the transcription of fibrogenic and pro-tumorigenic genes via canonical TGF-β1/Smad2/3 signaling, thereby contributing to desmoplasia and tumor progression.^27^^,^^28^^,^^29^

### Glycosylation

While glycosylation in general has been demonstrated to play critical roles in cancer progression, including cellular adhesion, resistance to cell death, and metastatic potential, there is currently a lack of knowledge regarding mesothelin glycosylation.^30^^,^^31^ To address this knowledge gap, Duran et al. conducted a comprehensive analysis of the N-glycan structures of MSLN from pancreatic ductal adenocarcinoma (PDAC) cell lines and tissues. Their findings revealed full occupancy of the three N-glycosylation sites (N388, N488, and N515) and a structure predominantly corresponding to a complex core fucosylated-N-glycan with terminal sialylation on MSLN from the cell-conditioned media.^32^ By contrast, a decrease of core-fucosylated glycoforms of MSLN was observed in both serum and tissues from PDAC patients. This observation was replicated by Zhan et al. who demonstrated that N-glycan fucosylation was predominant on soluble MSLN (sMSLN) from a pancreatic cancer cell line, while in ascites, sMSLN exhibited heavy sialylation and moderate fucosylation.^16^ These two studies indicate a shift in the glycoforms of MSLN during the progression of pancreatic cancer, with a decrease in core-fucosylated glycoforms and an increase in highly sialylated and moderately core-fucosylated glycoforms.

Fujihira and colleagues have demonstrated that the glycosylation profile of MSLN in epithelial mesothelioma differs from that of MSLN in normal mesothelial cells, thereby supporting a potential role for glycosylation in the malignant transformation of cells.^33^

### Shedding

In cancer, MSLN shedding is mediated by upregulated and activated enzymes, such as members of the ADAM, MMP, TACE, and BACE families.^15^ These enzymes cleave MSLN at different sites in the C-terminal region close to the cell surface (Figure 1C). The action of these proteases contributes to the elevated shedding rate that appears to vary according to the cancer type and the tumor stage.^34^ The presence of sMSLN in the bloodstream has been demonstrated in patients diagnosed with mesothelioma and ovarian cancer. Conversely, patients diagnosed with pancreatic adenocarcinoma exhibit minimal or no increase in serum mesothelin concentration. A recent study has demonstrated, employing PDAC models, that shed mesothelin is sequestered within the tumor microenvironment (TME). The authors of the study postulated that this phenomenon is associated with collagen density and elevated MUC16 expression.^34^

### Prognostic value

The sMSLN and the membrane-bound MSLN (mbMSLN) have been the focus of studies exploring their potential as biomarkers for various cancers, particularly for mesothelioma, pancreatic cancer, and ovarian cancer. However, as highlighted in an extensive review by Hagerty and Takabe, the heterogeneity of study designs, outcomes, and protocols complicates the interpretation of their clinical relevance.^35^ Furthermore, the co-expression status of the ligand MUC16 is rarely addressed in studies examining the prognostic value of MSLN. This information could offer valuable insights into clinicopathological implications that could influence prognosis, as MUC16 co-expression has been linked to accelerated tumor growth, metastasis, and poor prognosis. Recently MSLN-MUC16 co-expression has been associated to tumor aggressiveness and poor prognosis in patients with PDAC,^36^^,^^37^^,^^38^ endometrial serous carcinoma,^39^^,^^40^ perihilar bile duct carcinoma,^41^ and luminal-type breast cancer.^42^ Regarding the use of MSLN as a diagnostic biomarker, it has been demonstrated that neither elevated levels of circulating mesothelin nor the expression of membrane-bound mesothelin, in isolation, are adequate for establishing a reliable diagnosis of cancer. However, when considered in conjunction with other markers, MSLN can serve as an indication of cancer pathology and provide a compelling rationale for further investigations.^43^

**Figure 2:**
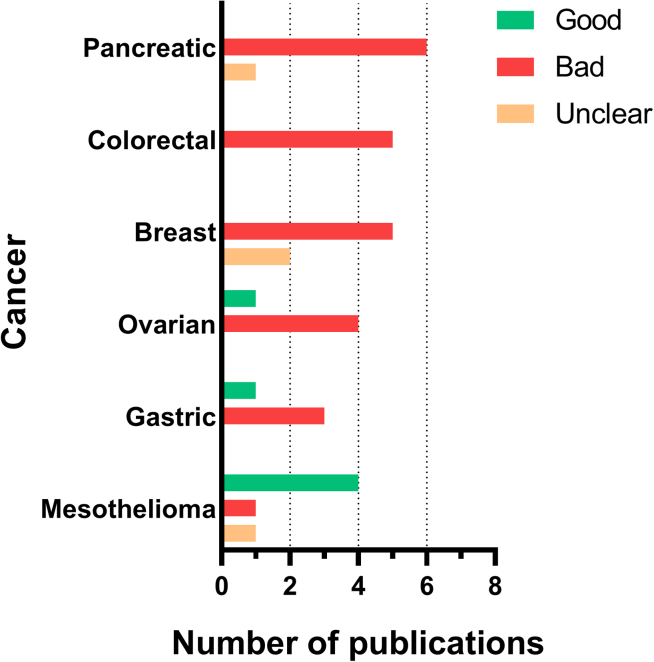
Prognostic value regarding MSLN overexpression in cancers The cancers presented are those for which at least three studies have demonstrated the prognostic value of MSLN. Cancers are listed in descending order of publication demonstrating a correlation between MSLN level expression and bad prognosis: pancreatic,^36^^,^^37^^,^^38^^,^^44^^,^^45^^,^^46^^,^^47^ colorectal,^48^^,^^49^^,^^50^^,^^51^^,^^52^ breast,^42^^,^^53^^,^^54^^,^^55^^,^^56^^,^^57^^,^^58^ ovarian,^59^^,^^60^^,^^61^^,^^62^^,^^63^ gastric,^64^^,^^65^^,^^66^^,^^67^ and mesothelioma.^18^^,^^68^^,^^69^^,^^70^^,^^71^^,^^72^

Several prognostic survival studies have been conducted on various MSLN-positive cancers, with the aim of determining the prognostic significance of soluble and/or membrane MSLN (Figure 2). A significant number of these studies have validated the correlation between elevated MSLN expression in immunohistochemistry and unfavorable prognosis in multiple cancers, including malignant pleural mesothelioma (MPM),^68^ ovarian cancer,^59^^,^^60^ and triple-negative breast cancer (TNBC).^53^

The cellular localization of MSLN has been demonstrated to possess potential prognostic value, with luminal/membranous localization of MSLN being associated with a more unfavorable prognosis in pancreatic cancers and extrahepatic bile duct cancers when compared with cytoplasmic localization.^19^ A recent observation by a Japanese research team has revealed a similar finding, demonstrating that membrane MSLN positivity in luminal-type breast cancers appears to be associated with a poor prognosis and an elevated risk of recurrence.^42^^,^^54^

In the context of colorectal cancer, elevated MSLN expression has been associated with chemoresistance, peritoneal metastases, and a lower 3-year overall survival rate.^48^^,^^49^

The presence of sMSLN in the bloodstream has not yielded satisfactory prognostic results for pancreatic cancers. This is due to the fact that serum levels of mesothelin do not differentiate between stages of the disease nor reflect tumor burden.^73^

Conversely, a systematic review of 20 studies regarding MPM demonstrated that a decrease in serum MSLN levels correlates with a positive response to treatment. This suggests that measuring serum MSLN could enable the monitoring of disease progression, risk of recurrence after surgery, and response to systemic therapy.^74^ Data from a clinical trial for a specific CAR-T cell -MSLN immunotherapy approach in malignant pleural mesothelioma (NTC02414269) presented at the American Society of Clinical Oncology (ASCO) and the American association for cancer research (AACR) in 2019 point in the same direction. The observed decrease in serum sMSLN levels is associated with tumor regression and persistence of CAR-T cells.^75^

It is noteworthy that a recent publication reported the potential predictive value of MSLN regarding therapies.^76^ The study demonstrated a significant correlation between MSLN and both cetuximab and gefitinib sensitivity in pancreatic cancer.

### Role in cancer

**Figure 3:**
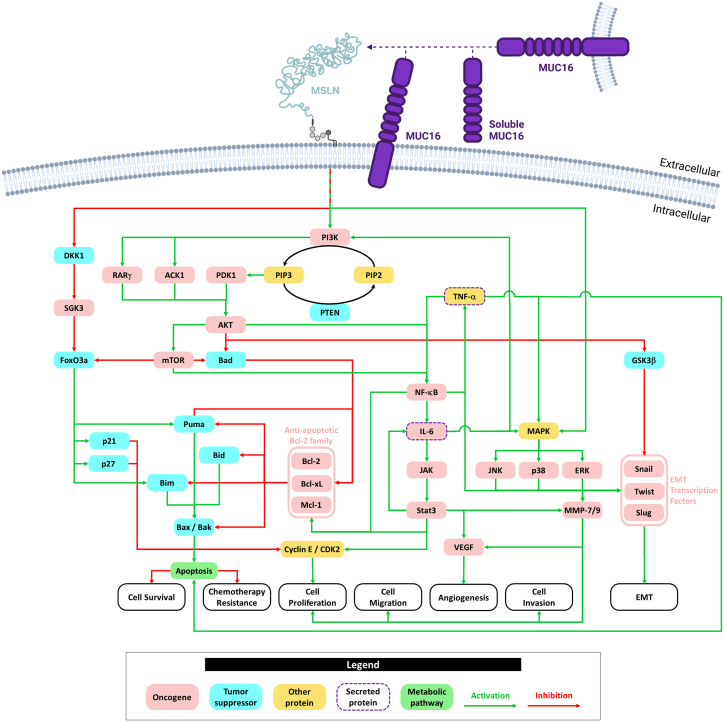
Schematic representation of MSLN-related metabolic pathways in cancer Regarding apoptosis pathways, under PI3K activation, mesothelin induces AKT phosphorylation and regulates RARG and TNK2 (encoding RARγ and ACK1), known to phosphorylate and activate AKT, promoting cell survival and disease progression in pancreatic and hepatocellular carcinoma.^78^^,^^79^ AKT activation, through RARγ, ACK1, and PDK1, leads to the inhibition of the transcription factor Forkhead box O3a (FoxO3a) through mTOR.^80^ This inhibition is emphasized by MUC16-MSLN interaction leading to SGK3/FOXO3 pathway activation, as DDK1 is inhibited stopping SGK3 kinase inhibition, a FoxO3a inhibitor.^81^ Phosphorylated FoxO3a is translocated to the cytoplasm, ubiquitinated and degraded.^82^ Thus, two BH3-only sensitizers are inhibited: Bad, by AKT and mTOR, and Puma, no longer activated by FoxO3a.^83^ In healthy condition, Bad and Puma inhibit anti-apoptotic multidomain Bcl-2 proteins (e.g., Bcl-2, Bcl-xL, and Mcl-1). In MSLN-positive cancers, these proteins are no longer inhibited. Furthermore, anti-apoptotic Bcl-2 proteins are activated by NF-κB and Stat3 both overactivated in MSLN^+^ cancers. In normal condition, the BH3-only activator family, made up of Bim, Bid, and Puma, promote Bax/Bak heterodimerization, leading to death cell.^83^ In MSLN-positive cancers, Bim is partly activated by FoxO3a, which is inhibited by MSLN, and the BH3-only activator family is inhibited by the now activated anti-apoptotic Bcl-2 proteins.^83^ Thus, MSLN in cancer decreases apoptosis, thereby promoting cell survival. Furthermore, the expression level of MSLN has been demonstrated to be correlated with chemotherapy resistance.^48^^,^^59^^,^^61^^,^^84^ This phenomenon can be explained by the inhibition of the apoptosis pathway. Regarding cell proliferation pathways, AKT and mTOR activate NF-κB pathway, leading to the production of IL-6 known to activate AKT through PI3K, generating previously discussed events.^85^^,^^86^ In pancreatic cancers, mesothelin expression level correlates with IL-6 expression.^87^ NF-κB also generates a self-activation loop through TNF-α and promote anti-apoptotic Bcl-2 proteins.^88^ Despite TNF-α overexpression, a protein considered as inducing death cells, tumor cells might survive using various mechanisms and may acquire resistance.^89^ Autocrine IL-6 binds its receptor, inducing JAK phosphorylation, leading to Stat3 activation through homodimerization mediated by subunit phosphorylation.^85^^,^^90^ The IL-6/Stat3 pathway is renown in cancer for promoting cell survival, proliferation, and migration.^91^^,^^92^ Stat3 acts as a transcription factor for oncogenes like IL-6, anti-apoptotic Bcl-2 proteins, and cyclin E, which, through the cyclin E/CDK2 complex, induces cell proliferation.^93^ Under normal condition, cyclin E/CDK2 complex is closely regulated by p21 and p27, both activated by FoxO3a, inhibited in MSLN-positive cancers.^94^ Cell proliferation, cell migration, and cell invasion pathways appear closely related to MMP-7 and MMP-9.^37^^,^^95^ Mesothelin directly activates MAPK cascades and indirectly through IL-6 and TNF-α, leading to ERK, JNK, and p38 activation.^88^^,^^96^^,^^97^ These three proteins, along with Stat3, promote MMP-7, leading to cell migration and invasion.^37^^,^^98^ Additionally, cases of increased angiogenesis through MSLN have been reported, notably in PDAC.^99^ IL-6 seems to play a role in angiogenesis mediated by MSLN as a correlation between IL-6 and vascular endothelial growth factor (VEGF) expression in cancers is well established.^100^ While no study has clearly proven a pathway involving MSLN, IL-6, Stat3, HIF-1α, and VEGF, the Stat3, HIF-1α, and VEGF correlation is well established in many cancers, suggesting MSLN could regulate angiogenesis through this same pathway.^101^ Moreover, MMP-7 is also increasing angiogenesis, at least in part, by stimulating the proliferation of VEGF.^98^ Finally, MSLN has been demonstrated to induce EMT by activating EMT transcription factors.^84^^,^^102^ This mechanism is known in cancer to be activated through AKT, which inhibits GSK3β and activates NF-κB, and through ERK, JNK, and p38.^103^ ACK1, activated CDC42-associated kinase 1; Bad, Bcl-2-associated agonist of cell death; Bak, Bcl-2 antagonist/killer; Bax, Bcl-2-associated X-protein; Bcl-2, B cell lymphoma-2; Bcl-xL, B cell lymphoma-extra large; Bid, BH3-interacting domain death agonist; Bim, Bcl-2-interacting mediator of cell death; CDK2, cyclin-dependent kinase 2; DKK1, Dickkopf 1; EMT, epithelial-mesenchymal transition; ERK, extracellular-signal-regulated kinase; FoxO3a, Forkhead box class O 3a; GSK3β, glycogen synthase kinase-3 β; IL-6, interleukin 6; JAK, Janus kinase; JNK, c-Jun N-terminal kinase; MAPK, mitogen-activated protein kinase; Mcl-1, myeloid cell leukemia-1; MMP-7/9, matrix metalloproteinase-7/matrix metalloproteinase-9; mTOR, mammalian target of rapamycin; NF-kB, nuclear factor kappa-light-chain-enhancer of activated B cells; PDK1, phosphoinositide-dependent protein kinase-1; PI3K, phosphoinositide 3-kinase; PIP2, phosphatidylinositol 4,5-bisphosphate; PIP3, phosphatidylinositol-3,4,5-trisphosphate; PTEN, phosphatase and tensin homolog; Puma, p53 upregulated modulator of apoptosis; RARγ, retinoic acid receptor γ; SGK3, serum and glucocorticoid-inducible protein kinase 3; Stat3, signal transducer and activator of transcription 3; TNF-α, tumor necrosis factor alpha.

Preclinical and clinical data suggest an active role of MSLN overexpression in the invasive and aggressive phenotypes of MSLN-positive tumors. It is generally accepted that MSLN overexpression promotes tumor cell survival, proliferation, and migratory and invasive capacities and contributes to chemoresistance. However, the precise mechanisms by which MSLN exerts its effects and its interactions with the TME are not yet fully understood. One hypothesis suggests that MSLN may interact with various signaling transmembrane proteins within lipid rafts, a phenomenon observed with GPI-anchored proteins.^77^ It appears that MSLN can function in a MUC16-dependent or MUC16-independent manner (Figure 3). However, distinguishing between these two modes of action is challenging due to the lack of systematic assessment of MUC16 status in studies. Additionally, the distinction between the soluble and membrane forms of mesothelin is not always clear in existing literature.

MSLN overexpression has been demonstrated to regulate multiple signaling pathways identified as contributing to cancer progression (Figure 3). This review does not aim to provide an exhaustive overview of all these pathways. Instead, it will offer an overview of some of the pathophysiological implications observed in different types of cancer.

In pancreatic cancer, MSLN-dependent nuclear factor κB (NF-κB) pathway activation has been observed to result in elevated levels of interleukin-6 (IL-6), which, in turn, have been found to be responsible for the activation of anti-apoptotic factors, such as the Bcl-2 family.^87^^,^^89^^,^^93^ In mesothelioma and pancreatic cancer, mitogen-activated protein kinase (MAPK) pathway activation through MSLN has been shown to promote cell migration and invasion by facilitating the expression and activation of matrix metalloproteinase (MMP)-7 and MMP-9, which are metalloproteinases implicated in the degradation of the extracellular matrix.^37^^,^^95^ In pancreatic cancer, independently of MUC16, MSLN contributes to the development of peritoneal metastasis by enhancing angiogenesis, cell proliferation, and invasion.^99^ In breast cancer cell lines, ectopic expression of MSLN facilitates anchorage-independent cell growth *in vitro* and induces resistance to anoikis.^104^

Recent studies have demonstrated that the inactivation of MSLN in various cancer cell lines disrupts the expression of several genes associated with the epithelial-mesenchymal transition (EMT). These results suggest that MSLN promotes the EMT and stem cell properties by enhancing the expression of EMT regulators (snail, twist, slug, ABCG2) and reducing the expression of mesenchymal markers (E-cadherin, caveolin-2, cytokeratins, and claudins).^84^^,^^102^ *In vivo*, genetic knockdown of MSLN has been demonstrated to decrease tumor growth and metastasis, while mesothelin overexpression in a non-cancerous cell line has been shown to induce a malignant phenotype, arguing in favor of an oncogenic role for mesothelin.^102^

In ovarian cancers, particularly in high-grade serous carcinoma, a correlation has been observed between MSLN expression levels and chemoresistance.^59^^,^^61^ A close association has also been established between chemotherapy resistance and the level of MSLN expression in malignant pleural mesothelioma and pancreatic cancers.^68^^,^^84^

MSLN has also been identified as a potential contributor to the progression of lung cancer, particularly through its role in facilitating the disruption of the blood-brain barrier (BBB) in non-small cell lung cancer (NSCLC).^105^

Beyond its oncogenic roles, recent studies have demonstrated a correlation between high MSLN expression and the remodeling of the tumor immune landscape.^106^ Bioinformatics approaches have demonstrated a positive correlation between high MSLN expression and immune infiltration in ovarian cancers, including Th17, dendritic cells, and natural killer (NK) cells.^61^ In microsatellite-stable colorectal tumors, MSLN overexpression promotes immune checkpoint expression (PD-1, LAG-3, TIM-3), immune-related marker expression (PD-L1), immune cell infiltration, and the activation of pro-tumoral pathways (TGF-β and IL-6/JAK/Stat3).^107^ In PDAC, MSLN expression has been associated with reduced cytotoxic T cell infiltration, increased PD-L1 expression in the stroma, and transcriptional features of T cell exhaustion, suggesting an immune-excluded phenotype.^108^ Still in PDAC, sMSLN promotes the polarization of macrophages into an immunosuppressive phenotype (CD106^+^), which supports tumor cell growth, neutrophil recruitment, and the spread of metastasis to the lungs.^109^ Most studies indicate that MSLN expression negatively contributes to the remodeling of the TME, by promoting immunosuppressive features in several cancers. However, a recent study reports that the role of MSLN may be context-dependent, as in MPM its expression appears to reshape the immune landscape of the TME by promoting the recruitment of CD8^+^ T cells and CD68^+^ macrophages, while increasing the abundance of type I collagen fibers.^110^

## Strategies in clinical trials targeting mesothelin

As described above, MSLN has emerged as a promising tumor-associated antigen (TAA). The field of mesothelin-targeted therapies has undergone continuous evolution over the past 25 years, encompassing the following three categories: vaccine, antibody-based immunotherapy, and cell therapy. However, despite the extensive research conducted in this field, no MSLN-targeted treatment has yet received the approval for clinical utilization from the Food and Drug Administration (FDA). In the following section, recent clinical trial data related to these approaches are compiled.

### Vaccines

#### Listeria monocytogenes-based vaccines: CRS-207 and JNJ-757

CRS-207, developed by Aduro Biotech, is an attenuated strain of *Listeria monocytogenes* engineered to express the human mesothelin within the cytosol of infected antigen-presenting cells (APCs).^12^^,^^111^ This strain has been genetically modified through the deletion of two virulence genes, significantly reducing its pathogenicity while preserving its capacity to elicit robust immune responses.^112^

Since the first trial in 2007 with CRS-207, 11 clinical trials have been registered on ClinicalTrials.gov: eight phase 2, one phase ½, and two phase 1 (Table 1).^116^ CRS-207 has been evaluated both as monotherapy and in combination with different therapies such as immune checkpoint inhibitors (ICIs) (ipilimumab, nivolumab, and pembrolizumab), chemotherapies (epacadostat, an oral inhibitor of the IDO1 enzyme, cisplatin, cyclophosphamide, and pemetrexed), tadalafil, or vaccine (GVAX pancreas vaccine). The drug has been studied in patients with various types of cancer. In a clinical trial for patients with advanced MPM (NCT01675765), the combination treatment of CRS-207 and chemotherapy demonstrated a higher overall response rate compared to chemotherapy alone.^117^ By contrast, the combination treatment of CRS-207 and pembrolizumab in patients with MPM exhibited no clinical activity, resulting in the cessation of the clinical development of CRS-207 for this indication.^115^ Despite this discontinuation, mesothelin-targeted CRS-207 in combination with immune checkpoint inhibitors, dioxygenase (IDO) inhibitors, and CY/GVAX is currently being evaluated in other indications, notably in patients with pancreatic cancers.^118^

**Table 1:** MSLN-targeted vaccines in clinical trials

Drug name	NCT number	Phase	Completion date	Enrollment	Sponsor	Study	Tumor type	Study outcomes	Reference
CRS-207	NCT05014776	II	estimated 2026-04-03	17	Sidney Kimmel Comprehensive Cancer Center at Johns Hopkins	evaluation of safety and efficacy of CRS-207 in combination with pembrolizumab, ipilimumab, and tadalafil *despite the use of CRS-207, this clinical trial is not focused on MSLN*	PDAC	**posology:** CRS-207 1 × 10^9^ CFU i.v. on day 2 of cycles 1–6 (3-week cycles) + pembrolizumab 200 mg i.v. on day 1 of cycles 1–6 + ipilimumab 50 mg i.v. on day 1 of cycles 1, 3, and 5 + tadalafil 20 mg po. on days 3–21 of cycles 1–6 **safety:** N/A **efficacy:** N/A	Gross et al.^113^
	NCT03006302	II	2024-08-05	41	Sidney Kimmel Comprehensive Cancer Center at Johns Hopkins	evaluation of safety, tolerability, and efficacy of CRS-207 in combination with epacadostat and pembrolizumab with or without CY/GVAX *despite the use of CRS-207, this clinical trial is not focused on MSLN*	pancreatic	**posology:** CRS-207 1 × 10^9^ CFU i.v. on day 2 of cycles 3–6 (3-week cycles). (For epacadostat, pembrolizumab, cyclophosphamide, and GVAX pancreas vaccine details: NCT03006302) **safety (*n* = 40):** 40% of patients experienced SAEs **efficacy (*n* = 26):** OS: 46% of patients survived more than 6 months	–
	NCT03190265	II	2022-08-23	61	Sidney Kimmel Comprehensive Cancer Center at Johns Hopkins	evaluation of safety and efficacy of nivolumab and ipilimumab in combination with either sequential administration of CY/GVAX followed by CRS-207 or with administration of CRS-207 alone *despite the use of CRS-207, this clinical trial is not focused on MSLN*	pancreatic	**posology:** CRS-207 1 × 10^9^ CFU i.v. on day 2 of cycles 3–6 (3-week cycles). (For nivolumab, ipilimumab, cyclophosphamide, and GVAX pancreas vaccine details: NCT03190265) **safety:** without CY/GVAX, 78% of patients experienced SAEs and 37% experienced grade ≥3 TRAEs. With CY/GVAX, 80% of patients experienced SAEs and 33% experienced grade ≥3 TRAEs **efficacy nivolumab + ipilimumab + CRS-207 (*n* = 30):** OR: CR + PR 7% **efficacy nivolumab + ipilimumab + CY/GVAX + CRS-207 (*n* = 31):** OR: CR + PR 0%	–
	NCT03122548∗	II	2018-01-31 (terminated due to low enrollment and lack of clinical activity in other CRS-207 studies)	5	Aduro Biotech	evaluation of safety and efficacy of CRS-207 in combination with pembrolizumab	gastric, GEJ, and esophageal	**posology:** CRS-207 1 × 10^9^ CFU i.v. on day 2 of cycle 1 (3-week cycles), day 1 of cycles 2–4 and then on day 1 every 6 weeks + pembrolizumab 200 mg i.v. on day 1 of each 3-week cycle **safety (*n* = 5):** 80% of patients experienced SAEs **efficacy (*n* = 5):** OS (*n* = 5): 2.5 months. PFS (*n* = 5): 1.2 months. OR (*n* = 3): CR 0%, PR 0%, SD 0%, and PD 100%	Kelly et al.^114^
	NCT03175172∗	II	2018-01-31 (terminated due to low enrollment and lack of clinical activity)	10	Aduro Biotech	evaluation of safety and efficacy of CRS-207 in combination with pembrolizumab *despite the use of CRS-207, this clinical trial is not focused on MSLN*	MPM	**posology:** CRS-207 1 × 10^9^ CFU i.v. on day 2 (3-week cycles) + pembrolizumab 200 mg i.v. on day 1. If tolerated, pembrolizumab and CRS-207 were administered on the same day for subsequent cycles. After 4 cycles, pembrolizumab was administered on day 1 every 3 weeks and CRS-207 once every 6 weeks **safety (*n* = 10):** 40% of patients experienced SAEs **efficacy (*n* = 10):** OS (*n* = 10): 2.8 months. PFS (*n* = 10): 1.4 months. OR (*n* = 9): CR 0%, PR 10%, SD 11%, and PD 89%	Alley et al.^115^
CRS-207	NCT02243371	II	2017-07-21	93	Sidney Kimmel Comprehensive Cancer Center at Johns Hopkins	evaluation of safety and efficacy of CY/GVAX followed by CRS-207 with or without nivolumab *despite the use of CRS-207, this clinical trial is not focused on MSLN*	pancreatic	**posology:** CRS-207 1 × 10^9^ CFU i.v. on day 2 of cycles 3–6 (3-week cycles) + cyclophosphamide 200 mg/m^2^ i.v. on day 1 of cycles 1 and 2 + GVAX 5 × 10^8^ cells in 6 i.d. injections on day 2 of cycles 1 and 2, w/ or w/o nivolumab 3 mg/kg i.v. on day 1 of cycles 1–6 **safety (*n* = 93):** 12% of patients treated with CY/GVAX + CRS-207 experienced grade ≥3 TRAEs (*n* = 42). 35% of patients treated with CY/GVAX + CRS-207 + nivolumab experienced grade ≥3 TRAEs (*n* = 51) **efficacy CY/GVAX + CRS-207 (*n* = 42):** OS: 6.1 months. PFS: 2.2 months. OR: CR 0%, PR 2%, SD 7%, and PD 67% **efficacy CY/GVAX + CRS-207 + nivolumab (*n* = 51):** OS: 5.9 months. PFS: 2.2 months. OR: CR 0%, PR 2%, SD 12%, and PD 69%	Tsujikawa et al.^118^
	NCT01417000	II	2017-02-10	93	Aduro Biotech	evaluation of safety and efficacy of CY/GVAX in combination with CRS-207 and CY/GVAX alone	PDAC	**posology:** in CY/GVAX alone condition, the treatment was on weeks 1, 4, 7, 10, 13, and 16. In combination condition, CY 200 mg/m^2^ i.v. on day 1 of weeks 1 and 4 + GVAX 1 × 10^8^ cells i.d. on day 2 of weeks 1 and 4 + CRS-207 1 × 10^9^ CFU i.v. on day 1 of weeks 7, 10, 13, and 16 **safety:** in CY/GVAX alone condition, 4 grade ≥3 AEs occurred (*n* = 29). In combination condition, 29 grade ≥3 AEs occurred (*n* = 61) **efficacy CY/GVAX alone (*n* = 29):** OS: 3.9 months. OR: SD 24% **efficacy CY/GVAX + CRS-207 (*n* = 61):** OS: 6.1 months. OR: SD 31%	Le et al.^192^
	NCT02004262	II	2016-08-23	303	Aduro Biotech	evaluation of safety and efficacy of CRS-207 in combination with CY/GVAX vs. CRS-207 alone vs. single-agent chemotherapy *despite the use of CRS-207, this clinical trial is not focused on MSLN*	pancreatic	**posology:** CRS-207 1 × 10^9^ CFU i.v. on day 1 of weeks 7, 10, 13, and 16. (For cyclophosphamide, GVAX pancreas vaccine, gemcitabine, capecitabine, fluorouracil, leucovorin, irinotecan, and erlotinib details: NCT02004262) **safety (*n* = 169):** in the below order of treatment conditions, 47%, 37%, and 28% of patients experienced SAEs. Most frequently reported AEs in all treatment groups were chills, pyrexia, fatigue, and nausea. No treatment-related deaths occurred **efficacy single-agent chemotherapy (*n* = 43, primary cohort):** OS: 4.7 months. PFS: 2.1 months **efficacy CRS-207 alone (*n* = 58, primary cohort):** OS: 5.2 months. PFS: 2.1 months **efficacy CRS-207 + CY/GVAX (*n* = 68, primary cohort):** OS: 4.2 months. PFS: 2.3 months	Le et al.^193^
CRS-207	NCT02575807∗	I/II	2018-05-08 (terminated due to low enrollment and lack of clinical activity)	35	Aduro Biotech	evaluation of safety and efficacy of CRS-207 alone and in combination with epacadostat and/or pembrolizumab *despite the use of CRS-207 this clinical trial is not focused on MSLN*	ovarian, fallopian and peritoneal	**posology:** CRS-207 1 × 10^9^ CFU i.v. Q3W for 6 cycles, then Q6W (For epacadostat, and pembrolizumab details: NCT02575807) **safety (*n* = 32):** In combination with CRS-207, epacadostat RP2D was determined as 300 mg po. All patients included, 44% experienced SAEs **efficacy epacadostat 300 mg (*n* = 28):** OS (*n* = 28): 2.1 (CRS-207 + epacadostat + pembrolizumab, *n* = 1) to 11.3 months (CRS-207 alone, *n* = 8). PFS (*n* = 28): 1.2 (CRS-207 + epacadostat + pembrolizumab, *n* = 1) to 1.8 months (CRS-207 alone and CRS-207 + epacadostat, *n* = 8 and *n* = 16). OR (*n* = 24): All patients combined the best response was SD (SD 31% with CRS-207 + epacadostat, *n* = 13)	–
	NCT01675765	I	2019-08-19	60	Aduro Biotech	evaluation of safety and efficacy of CRS-207 in combination with pemetrexed and cisplatin w/ or w/o cyclophosphamide	MPM	**posology:** CRS-207 1 × 10^9^ CFU on weeks 1, 3, 23, 26 and every 8 weeks from week 34 + pemetrexed 500 mg/m^2^ and cisplatin 75 mg/m^2^ on weeks 5, 8, 11, 14, 17, and 20 (up to six 3-week cycles), w/ or w/o cyclophosphamide 200 mg/m^2^ on weeks 1, 3, 23, and 26 **safety (*n* = 60):** 39% of patients treated with CRS-207 + pemetrexed + cisplatin experienced SAEs (*n* = 38). 50% of patients treated with CRS-207 + pemetrexed + cisplatin + cyclophosphamide experienced SAEs (*n* = 22) **efficacy CRS-207 + pemetrexed + cisplatin (*n* = 21):** OR: CR 3%, PR 53%, SD 14%, PD 3% and NE 3% **efficacy CRS-207 + pemetrexed + cisplatin + cyclophosphamide (*n* = 36):** OR: CR 0%, PR 52%, SD 38%, and PD 10%	Hassan et al.^194^
	NCT00585845	I	2009-02-01	17	Anza Therapeutics	evaluation of safety, tolerability, and pharmacokinetics of CRS-207	solid	**posology:** CRS-207 1 × 10^8^ to 1 × 10^10^ CFU i.v. up to 4 administrations every 3 weeks **safety (*n* = 17):** MTD was determined to be 1 × 10^9^ CFU. At the MTD, 100% of patients experienced grade 4 lymphopenia, 33% experienced grade 3 hypophosphatemia and 17% experienced grade 3 elevated gamma-glutamyl transferase (*n* = 6) **efficacy CRS-207 1 × 10**^**9**^**CFU (*n* = 6):** OS: 33% of patients survived more than 15 months. OR: CR 0%, PR 0%, SD 33%, and PD 67%	Le et al.^116^
JNJ-757 (JNJ-64041757)	NCT03371381∗	I/II	2018-10-09 (terminated due to lack of clinical benefit)	12	Janssen—Johnson & Johnson	evaluation of safety, pharmacokinetics, and efficacy of JNJ-757 in combination with nivolumab	NSCLC	**posology:** JNJ-757 1 × 10^9^ CFU i.v. Q4W + nivolumab 240 mg i.v. Q2W **safety (*n* = 12):** 50% of patients experienced grade ≥3 AEs, including two cases of treatment-related fatal pneumonitis **efficacy (*n* = 11):** OR: CR 0%, PR 0%, SD 36%, PD 45%, and NE 18%	Brahmer et al.^119^
Neoantigen DNA vaccine	NCT03122106∗	I	2022-08-13 (terminated due to loss of funding)	15	Washington University School of Medicine	evaluation of safety and efficacy of neoantigen DNA vaccine	pancreatic	**posology:** 2 injections (1 into each deltoid or lateralis) on days 1, 5, 9, 13, 17, and 21 **safety (*n* = 11):** no patients experienced SAE. No grade ≥3 AEs were reported **efficacy (*n* = 9):** 100% of patients analyzed by ELISPOT experienced a neoantigen-specific immune response	Panni et al.^120^
Neoantigen peptide vaccine	NCT03956056∗	I	2023-07-06 (terminated due to insufficient funding/staff)	12	Washington University School of Medicine	evaluation of safety and efficacy of neoantigen peptide vaccine in combination with poly-ICLC	pancreatic	**posology:** neoantigen peptide vaccine + poly-ICLC on days 1, 4, 8, 15, 22, 50, and 78 sc **safety (*n* = 10):** 10% of patients experienced SAEs **efficacy (*n* = 9):** 100% of patients analyzed by ELISPOT experienced a neoantigen-specific immune responses	Panni et al.^120^

Discontinued clinical trials are shown with the symbol ∗. AE, adverse event; CFU, colony-forming units; CR, complete response; CY/GVAX, cyclophosphamide + GVAX pancreas vaccine; GEJ, gastroesophageal junction; i.d., intradermal; i.v., intravenous; MPM, malignant pleural mesothelioma; MTD, maximum tolerated dose; NE, not evaluated; NSCLC, non-small-cell lung cancer; OR, overall response; OS, overall survival; PD, progressive disease; PDAC, pancreatic ductal adenocarcinoma; PFS, progression-free survival; po., per os; PR, partial response; Q2W, once every 2 weeks; Q3W, once every 3 weeks; Q4W, once every 4 weeks; Q6W, once every 6 weeks; RP2D, recommended phase 2 dose; SAE, serious adverse event; sc., subcutaneous; SD, stable disease; TRAE, treatment-related adverse event.

A similar vaccine (JNJ-757), developed by Janssen, a Johnson & Johnson subsidiary, has undergone early phase trials, but the absence of clinical activity prompted its discontinuation.^119^

#### Neoantigen DNA and peptide vaccines

The Washington University School of Medicine has developed personalized cancer DNA- and peptide-based vaccines that incorporate prioritized neoantigens and mesothelin epitopes for patients with pancreatic cancers.^120^

Both approaches reached phase 1 clinical trials, demonstrating an acceptable toxicity and a neoantigen-specific immune response in all patients analyzed by ELISPOT (Table 1).^120^ However, despite the encouraging preliminary results, both trials were terminated due to insufficient funding.

### Antibody-based immunotherapies

**Figure 4:**
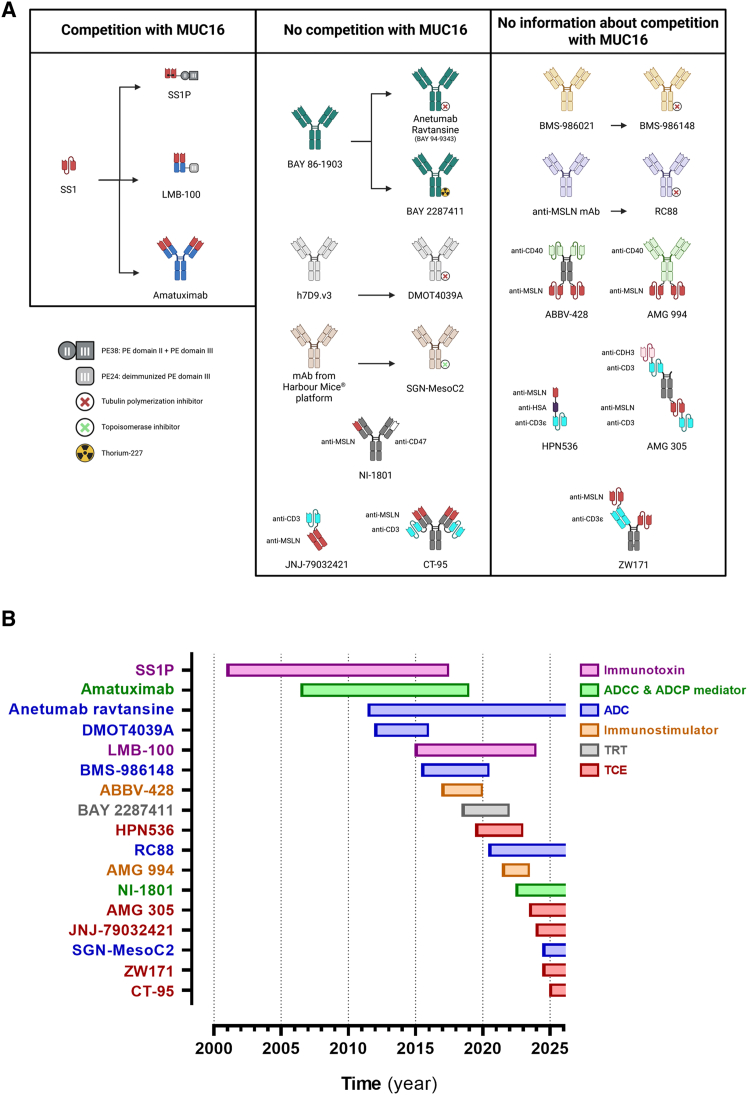
Schematic representation of antibody-based immunotherapies targeting MSLN that have advanced to the clinical trial phase, along with the duration of their clinical study (A) The molecules were separated based on the targeting epitope on MSLN. Regarding molecules that compete with MUC16, based on SS1 three molecules were engineered: SS1P (immunotoxin, dsFv conjugated to PE38), LMB-100 (immunotoxin, Fab conjugated to PE24), and amatuximab (ADCC and ADCP mediator, chimeric human antibody). Regarding molecules that do not compete with MUC16, based on BAY 86–1903, two molecules were engineered: BAY 94–9343 (ADC, payload: DM4) and BAY 2287 (TRT, radionuclide: ^227^Th). Based on h7D9.v3, DMOT4039A was engineered (ADC, payload: MMAE). Concerning SGN-MesoC2 (ADC, payload: SGD-12280) and NI-1801 (ADCC and ADCP mediator, bispecific), it is likely that the MSLN arm targets a membrane-proximal epitope on MSLN. JNJ-79032421 (TCE, bispecific) and CT-95 (TCE, bispecific) were engineered to target a membrane-proximal epitope on MSLN. For seven molecules no information was found about their competition with MUC16: BMS-986148 (ADC, payload: tubulysin), RC88 (ADC, payload: MMAE), ABBV-428 (immunostimulator, bispecific), AMG 994 (immunostimulator, bispecific), HPN536 (TCE, trispecific), AMG 305 (TCE, trispecific), and ZW171 (TCE, trispecific). (B) Representation of the clinical trial periods for the 17 anti-MSLN antibody-based immunotherapies. ADC, antibody-drug conjugate; ADCC, antibody-dependent-cell-mediated cytotoxicity; ADCP, antibody-dependent-cell-mediated phagocytosis; DM4, ravtansine; dsFv, disulfide-stabilized Fv; has, human serum albumin; ICE, immune cell-engager; MMAE, monomethyl auristatin E; MSLN, mesothelin; MUC16, mucin 16; PE, pseudomonas Eexotoxin A; SGD-12280, novel topoisomerase 1 inhibitor; TCE, T cell engager; TRT, targeted radionuclide therapy.

Currently, 17 antibody-based immunotherapies have advanced to the clinical research stage: two immunotoxins (iTOXs), two antibody-dependent cell-mediated cytotoxicity (ADCC) and antibody-dependent cell-mediated phagocytosis (ADCP) mediators, five antibody-drug conjugates (ADCs), two immunostimulators, one targeted radionuclide therapy (TRT), and five T cell engagers (TCEs) (Figure 4A).

The development of anti-MSLN treatment was initiated in 1998 under the direction of Ira Pastan. Ira Pastan’s team used a mesothelin expression plasmid to immunize mice. This approach led to the isolation of an anti-mesothelin single-chain variable fragment (scFv) known as SS scFv.^121^ Mutations were introduced in the complementarity determining region 3 (CDR3) of SS V_L_ to enhance its binding affinity, resulting in the variant named SS1.^122^ Notably, SS1 competes with MUC16 for MSLN binding.^25^ SS1 was regularly used to develop anti-MSLN drugs including SS1P, LMB-100, amatuximab, and even CAR-T cells (Figure 4B).^122^^,^^123^

#### Immunotoxins: SS1P and LMB-100

A recent review by Hagerty et al. provides a comprehensive summary of both pre-clinical and clinical data regarding MSLN-targeted immunotoxin (iTOX).^124^ The two iTOXs that advanced to the clinical trial stage are derived from the scFv SS1 and conjugated to truncated forms of *Pseudomonas* exotoxin A (PE) (Figure 4A). The mechanism of action of these iTOXs involves the impairment of cellular functions, inducing cell death through apoptosis.^125^ Following MSLN binding, the iTOX is internalized through receptor-mediated endocytosis. During intracellular trafficking, the cleaved toxin part induces the irreversible ADP ribosylation of elongation factor-2 (EF-2), which is crucial for protein elongation during translation.

SS1P (SS1(dsFv)PE38 or CAT-5001), developed by the National Cancer Institute (NCI), is the disulfide-stabilized SS1 fused to the truncated PE form known as PE38 (38 kDa).^122^^,^^126^ SS1P has been evaluated in clinical trials both as monotherapy and in combination with bevacizumab, cisplatin, carboplatin, cyclophosphamide, paclitaxel, pemetrexed, and pentostatin, mainly in patients with MPM or ovarian and pancreatic cancers (Table 2). Despite some evidence of anti-tumor effectiveness, reported clinical activity has been modest, and its development has faced critical challenges due to immunogenicity with the development of neutralizing anti-drug antibodies (ADA) against PE and toxicity.^129^

**Table 2:** MSLN-targeted antibody-based therapies in clinical trials

Drug type	Drug name	NCT number	Phase	Completion date	Enrollment	Sponsor	Study	Tumor type	Study outcomes	Reference
Immunotoxin	SS1P (SS1(dsFv)PE38 or CAT-5001)	NCT01362790	I/II	2017-08-07	55	National Cancer Institute	evaluation of safety, tolerability, and efficacy of SS1P in combination with pentostatin and cyclophosphamide	mesothelioma, pancreatic and lung adenocarcinoma	**posology:** SS1P 35 μg/kg i.v. on days 10, 12, and 14 for 1 cycle then on days 2, 4, and 6 for a maximum of 6 cycles + pentostatin + cyclophosphamide (regimen A). SS1P 35 μg/kg i.v. on days 18, 20, and 22 for 1 cycle then on days 6, 8, and 10 for a maximum of 6 cycles + pentostatin + cyclophosphamide (regimen B). (For pentostatin and cyclophosphamide details: NCT01362790) **safety depending on the regimen (*n* = 53):** at 35 μg/kg, 25%–100% patients experienced SAEs (*n* = 45). No SS1P-related grade 4 AEs reported. MTD was determined to be 25 μg/kg (*n* = 8) **efficacy depending on the regimen (*n* = 45):** OS: 4.2–29.3 months. PFS: 3.9–11.8 months. OR: CR 0%, PR 5%, SD 5%, PD 7% and NE 82%	Hassan et al.^127^
		NCT01445392	I	2016-10-03	24	National Cancer Institute	evaluation of safety, tolerability, pharmacokinetics, and efficacy of SS1P in combination with pemetrexed and cisplatin	MPM	**posology:** SS1P 25–55 μg/kg i.v. on days 1, 3, and 5 for 2 cycles (3-week cycle) + pemetrexed 500 mg/m^2^ i.v. on day 1 for up to 6 cycles + cisplatin 75 mg/m^2^ i.v. on day 1 for up to 6 cycles **safety:** MTD was determined to be 45 μg/kg. 100% of patients experienced AEs. No SS1P-related grade 4 AEs **efficacy (*n* = 24):** OS: 13.6 months. PFS: 6.0 months. OR: CR 0%, PR 50%, SD 13%, PD 21% and NE 17% **efficacy SS1P 45 μg/mg (*n* = 13):** OR: CR 0%, PR 77%, SD 8% and PD 15%	Hassan et al.^128^
		NCT01051934	I	2011-09-28	2	National Cancer Institute	evaluation of safety, tolerability, and pharmacokinetics of SS1P in combination with paclitaxel, carboplatin and bevacizumab	NSCLC	**posology:** N/A **safety:** N/A **efficacy:** N/A	–
		NCT00006981	I	N/A (publication 2009)	24	National Cancer Institute	evaluation of safety, tolerability, pharmacokinetics, and efficacy of SS1P	mesothelioma, ovarian and pancreatic	**posology:** 10 injections of SS1P 4 to 25 μg/kg/day i.v. **safety (*n* = 24):** MTD was determined to be 25 μg/kg/day. All patients experienced at least 1 AE. 13% of patients experienced grade ≥3 AEs **efficacy (*n* = 24):** OR: CR 0%, PR 4%, SD 50%, and PD 46%	Kreitman et al.^129^
		NCT00066651	I	N/A (publication 2007)	34	National Cancer Institute	evaluation of safety, tolerability, pharmacokinetics, and efficacy of SS1P	mesothelioma, ovarian and pancreatic	**posology:** with 6 injections QOD of SS1P: 8 to 25 μg/kg i.v. With 3 injections QOD of SS1P: 25 to 60 μg/kg i.v. **safety (*n* = 34):** MTD with 6 injections was determined to be 18 μg/kg/dose (*n* = 17) and with 3 injections to be 45 μg/kg/dose (*n* = 17). SS1P is well-tolerated with pleuritis as the DLT at the highest dose level **efficacy (*n* = 34):** OR: CR 0%, PR 12%, SD 56%, PD 29% and NE 3%	Hassan et al.^130^
Immunotoxin	SS1P (SS1(dsFv)PE38 or CAT-5001)	NCT00024674∗	I	2001-02-01 (withdrawn, continued by NCI NCT00006981)	0	INSYS Therapeutics	evaluation of safety and efficacy of SS1P	N/A	**posology:** 10-day continuous i.v. **safety:** N/A **efficacy:** N/A	–
		NCT00024687∗	I	2000-11-01 (withdrawn, continued by NCI NCT00006981)	0	INSYS Therapeutics	evaluation of safety, tolerability, pharmacokinetics and efficacy of SS1P	N/A	**posology:** 6 injections i.v. QOD **safety:** N/A **efficacy:** N/A	–
	LMB-100 (RG7787 or RO6927005)	NCT04027946∗	II	2023-12-22 (terminated due to slow accrual)	6	National Cancer Institute	evaluation of efficacy of LMB-100 followed by pembrolizumab	NSCLC	**posology:** LMB-100 140 μg/kg on days 1, 3, and 5 of 3-week cycles (up to 2) + 200 mg of pembrolizumab on day 1 of each subsequent 3-week cycles **safety (*n* = 3):** 0% of patients experienced grade ≥3 TRAEs. 100% of patients analyzed experienced SAEs. **efficacy (*n* = 3):** OS: 22.9 months. PFS: 3 months. OR: CR 0%, PR 0%, SD 67%, and PD 33%	Hassan et al.^131^
		NCT03644550∗	II	2020-11-02 (terminated due to principal investigator decision after FDA’s approval of nivolumab + ipilimumab as first line treatment for mesothelioma)	18	National Cancer Institute	evaluation of safety and efficacy of LMB-100 followed by pembrolizumab	Mesothelioma	**posology:** LMB-100 140 μg/kg i.v. on days 1, 3, and 5 of two 3-week cycles + pembrolizumab 200 mg i.v. Q3W from cycle 3 **safety (*n* = 18):** 78% of patients experienced SAEs **efficacy (*n* = 18):** OS (*n* = 18): 17.1 months. PFS (*n* = 18): 7.1 months. OR (*n* = 17): PR 18%	Hassan et al.^131^
		NCT02810418	I/II	2021-06-22	40	National Cancer Institute	evaluation of safety, tolerability, and efficacy of LMB-100 alone and in combination with nab-paclitaxel	PDAC	**posology:** LMB-100 65 or 100 μg/kg i.v. on days 1, 3, and 5 of 3-week cycles for 1–3 cycles w/ or w/o nab-paclitaxel 125 mg/m^2^ i.v. on days 1 and 8 **safety (*n* = 20):** with nab-paclitaxel, LMB-100 MTD was determined to be 65 μg/kg. Most common AEs were hypoalbuminemia (100%), edema (65%), fatigue (50%), hyponatremia (50%), and myalgia (45%) **efficacy LMB-100 65 μg/kg + nab-paclitaxel 125 mg/m**^**2**^**(*n* = 14):** OS: 5.3 months. OR: PR 7%. Although clinical activity was observed, the combination was not well tolerated	Alewine et al.^132^
		NCT05375825∗	I	2024-01-31 (withdrawn)	0	National Cancer Institute	evaluation of safety, tolerability, and pharmacokinetics of LMB-100	MPM	**posology:** LMB-100 1 mg/mL intrathoracic perfusion using a closed circuit and roller pump with a heat exchanger following maximal cytoreductive surgery **safety:** N/A **efficacy:** N/A	–
		NCT02798536	I	2022-04-21	21	National Cancer Institute	evaluation of safety, tolerability, pharmacokinetics, and efficacy of LMB-100 alone and in combination with nab-paclitaxel	Mesothelioma	**posology:** LMB-100 100 to 170 μg i.v. on days 1, 3, and 5 of each 3-week cycle (up to 2–4), w/ or w/o nab-paclitaxel 125 mg/m^2^ i.v. on days 1 and 8 of each 3-week cycle (up to 6) **safety (*n* = 21):** LMB-100 alone RP2D was determined to be 140 μg (*n* = 10). In combination with nab-paclitaxel, LMB-100 RP2D was determined to be 100 μg (*n* = 11). 0% of patients treated with LMB-100 at 140 μg experienced SAEs (*n* = 7). 33% of patients treated with LMB-100 at 170 μg experienced SAEs (*n* = 3). 36% of patients treated with LMB-100 at 100 μg + nab-paclitaxel experienced SAEs (*n* = 11) **efficacy LMB-100 140 μg (*n* = 7):** OS: 16.9 months. PFS: 2.7 months. OR: CR 0% and PR 0% **efficacy LMB-100 170 μg (*n* = 3):** OS: 33.5 months. PFS: 2.7 months **efficacy LMB-100 100 μg + nab-paclitaxel (*n* = 11):** OS: 10.1 months. PFS: 2.7 months. OR: CR 0% and PR 0%	Alewine et al.^132^
Immunotoxin	LMB-100 (RG7787 or RO6927005)	NCT04840615∗	I	2022-01-12 (terminated due to slow accrual)	2	National Cancer Institute	evaluation of feasibility and safety of LMB-100 intratumoral injections in combination with ipilimumab	mesothelioma	**posology:** LMB-100 400 μg itu. + ipilimubab itu. on days 1 and 4 each 3-week cycle (up to 4) **safety (*n* = 2):** 100% of patients experienced SAEs **efficacy (*n* = 2):** OS: 3.4 months. PFS: 1.4 months. OR: CR 0% and PR 0%	–
		NCT04034238	I	2021-11-19	19	National Cancer lInstitute	evaluation of safety, tolerability, pharmacokinetics, and efficacy of LMB-100 in combination with tofacitinib	mesothelioma, CHO, and PDAC	**posology:** LMB-100 65 to 140 μg/kg i.v. on days 4, 6, and 8 each 3-week cycles + tofacitinib 10 mg po. twice daily on days 1–10 **safety (*n* = 10):** with tofacitinib, LMB-100 MTD was determined to be 100 μg/kg on days 4, 6, and 8. At the MTD, 14% of patients experienced SAEs that may be related to LMB-100 (*n* = 7) **efficacy:** N/A	Pegna et al.^195^
		NCT03436732∗	I	2019-04-30 (terminated due to fatal occurrence attributed to one of the study drugs)	5	National Cancer Institute	evaluation of safety and tolerability of LMB-100 in combination with SEL-110	Mesothelioma	**posology:** LMB-100 100 or 140 μg/kg on days 1, 3, and 5 each 3-week cycles (up to 4) + SEL-110 on day 1 of each cycle at escalating doses **safety (*n* = 5):** the study was closed due to a fatal occurrence of pneumonitis attributed to one of the drugs **efficacy (*n* = 5):** OR: CR 0% and PR 0%	–
		NCT02317419∗	I	2015-08-01 (terminated due to the emerging benefit:risk ratio that did not justify continuing dosing patients)	15	Roche	evaluation of safety, tolerability, pharmacokinetics, and efficacy of LMB-100 alone and in combination with gemcitabine and nab-paclitaxel	Solid	**posology:** LMB-100 i.v. on days 1, 3, and 5 of each 3-4-week cycles, w/ or w/o gemcitabine + nab-paclitaxel on days 1, 8, and 15 of each 4-week cycles **safety:** N/A **efficacy:** N/A	Alewine et al.^132^
Antibody-drug conjugate	Anetumab ravtansine (BAY 94–9343)	NCT03587311	II	estimated 2025-10-21	96	National Cancer Institute	evaluation of safety, tolerability, pharmacokinetics, and efficacy of AR in combination with bevacizumab vs. paclitaxel in combination bevacizumab	HGOC	**Data cutoff date: 2024-11-27** **posology:** AR 2.2 mg/kg i.v. QW + bevacizumab 10 mg/kg i.v. Q2W. Paclitaxel 80 mg/m^2^ i.v. QW + bevacizumab 10 mg/kg i.v. Q2W **safety (*n* = 57):** with bevacizumab, AR RP2D was determined to be 2.2 mg/kg. 26 grade ≥3 TRAEs were reported with paclitaxel + bevacizumab (*n* = 29). 29 grade ≥3 TRAEs were reported with AR + bevacizumab (*n* = 28) **efficacy paclitaxel + bevacizumab (*n* = 29):** PFS: 12.7 months. OR: CR 0%, PR 66%, SD 20%, and PD 14% **efficacy AR + bevacizumab (*n* = 28):** PFS: 5.3 months. OR: CR 4%, PR 18%, SD 50%, and PD 28%	Alqaisi et al.^138^
		NCT03926143∗	II	2022-05-18 (terminated due to strategic company decisions)	9	Bayer	evaluation of long-term safety and efficacy of AR alone and in combination with gemcitabine	Solid	**posology:** N/A**safety (*n* = 9):** 100% of patients experienced TEAEs. 89% of patients experienced TRAEs. 22% of patients experienced TESAEs. 0% of patients experienced TRSAEs **efficacy (*n* = 9):** OS: 34.1 months	–
		NCT03023722	II	2019-12-11	18	Yale University	evaluation of safety and efficacy of AR	pancreatic	**posology:** AR 6.5 mg/kg i.v. Q3W. **safety (*n* = 18):** 61% of patients experienced SAEs **efficacy (*n* = 14):** TTP: 2.1 months. OR: CR 0%, PR 0%, SD 14%, and PD 86%	Chokshi and Hochster^196^
		NCT02610140	II	2019-09-06	248	Bayer	evaluation of safety, pharmacokinetics, and efficacy of AR vs. vinorelbine	MPM	**posology:** AR 6.5 mg/kg Q3W. Vinorelbine 30 mg/m^2^ Q3W **safety (*n* = 235):** 74% of patients treated with vinorelbine experienced grade ≥3 AEs and 15% experienced TESAEs (*n* = 72). 48% of patients treated with AR experienced grade ≥3 AEs and 7% experienced TESAEs (*n* = 163) **efficacy vinorelbine (*n* = 82):** OS (*n* = 82): 11.6 months. PFS (*n* = 82): 4.5 months. OR (*n* = 65): CR 0%, PR 9%, SD 77%, and PD 14% **efficacy AR (*n* = 166):** OS (*n* = 166): 9.5 months. PFS (*n* = 166): 4.3 months. OR (*n* = 146): CR 0%, PR 11%, SD 73%, and PD 16%	Kindler et al.^137^
		NCT02839681∗	II	2018-07-25 (terminated due to slow/insufficient accrual)	2	National Cancer Institute	evaluation of safety, tolerability, and efficacy of AR	Lung	**posology:** AR i.v. Q3W **safety (*n* = 2):** 50% of patients experienced an SAE (respiratory failure) **efficacy (*n* = 2):** OS: 1.7 months. PFS: 1.3 months. OR: CR 0% and PR 0%	–
		NCT03126630	I/II	estimated 2026-09-18	49	National Cancer Institute	evaluation of safety, tolerability, pharmacokinetics, and efficacy of AR in combination with pembrolizumab	MPM	**Data cutoff date: 2024-08-08** **posology:** pembrolizumab 200 mg i.v. w/ or w/o AR 6.5 mg/kg i.v. Q3W. (up to 1–2 years) **safety (*n* = 35):** 53% of patients treated with pembrolizumab alone experienced grade ≥3 AEs (*n* = 17) and 61% of patients treated with AR + pembrolizumab experienced grade ≥3 AEs (*n* = 18) **efficacy pembrolizumab alone (*n* = 17):** PFS: 3.9 months. OR: CR 0%, PR 6%, SD 29%, PD 53% and NE 12% **efficacy AR + pembrolizumab (*n* = 18):** PFS: 12.2 months. OR: CR 0%, PR 17%, SD 50%, PD 20% and NE 13%.	Mansfield et al.^139^
Antibody-drug conjugate	Anetumab ravtansine (BAY 94–9343)	NCT03455556∗	I/II	2020-01-07 (terminated due to slow accrual)	1	Mayo Clinic	evaluation of safety, tolerability, and efficacy of AR in combination with atezolizumab	NSCLC	**posology:** AR i.v. + atezolizumab i.v. Q3W **safety:** N/A **efficacy:** N/A	Hassan et al.^136^
		NCT03816358	I	estimated 2026-01-31	74	National Cancer Institute	evaluation of safety, tolerability, pharmacokinetic, and efficacy of AR in combination with nivolumab, w/ or w/o ipilimumab or gemcitabine	PDAC	**Data cutoff date: 2022-01-22** **posology:** AR 5.5 or 6.5 mg/kg i.v. on day 1 (3- to 4-week cycles) + nivolumab i.v. on day 8 of cycle 1 and on day 1 of subsequent cycles (up to 17), w/ or w/o ipilimumab i.v. on day 8 of cycle 1 and on day 1 of cycles 2–4 or gemcitabine on days 1 and 8**safety (*n* = 28):** 2 DLTs were reported for AR 6.5 mg/kg + nivolumab + ipilimumab. RP2D was determined to be 6.5 mg/kg. At 6.5 mg/kg, 5, 17 and 20% of patients experienced TRAEs, for AR + nivolumab, AR + nivolumab + ipilimumab, AR + nivolumab + gemcitabine, respectively **efficacy (*n* = 33):** following data correspond to AR + nivolumab (*n* = 11), AR + nivolumab + ipilimumab (*n* = 13), AR + nivolumab + gemcitabine (*n* = 9). OR: CR 0%, PR 0%, SD 18, 15, 89%, PD 64, 46, 0% and NE 18, 38, 11%	Spiliopoulou et al.^197^
		NCT03102320	I	2021-07-26	173	Bayer	evaluation of safety, tolerability, pharmacokinetics, and efficacy of AR alone and in combination with cisplatin or gemcitabine	gastric, NSCLC, TNBC, thymic, and PDAC	**posology:** AR 6.5 mg/kg i.v. on day 1 (3-week cycle), w/ or w/o cisplatin 25 mg/m^2^ i.v. or gemcitabine 1000 mg/m^2^ on day 1 and 8 (up to 6 cycles) **safety:** N/A **efficacy:** N/A	Adjei et al.^198^
		NCT02751918	I	2019-10-31	65	Bayer	evaluation of safety, tolerability, pharmacokinetics, and efficacy of AR in combination with PLD	ovarian, fallopian, and peritoneal	**posology:** AR 5.5 or 6.5 mg/kg i.v. + PLD 30 mg/m^2^ i.v. Q3W **safety (*n* = 65):** with PLD, AR MTD was determined to be 6.5 mg/kg (*n* = 9). No DLTs reported. More than 10% of patients experienced grade ≥3 AEs, fatigue (12%), neutrophil count decrease (11%) and neutropenia (11%) (*n* = 65) **efficacy (*n* = 65):** PFS: 5.0 months. OR: CR 2%, PR 26%, SD 43%, PD 17% and NE 12%. High MSLN expression increases treatment efficacy	Santin et al.^140^
		NCT02639091	I	2019-10-17	36	Bayer	evaluation of safety, tolerability, and pharmacokinetics of AR in combination with pemetrexed and cisplatin	mesothelioma and NSCLC	**Data cutoff date: 2017-06-12** **posology:** AR 5.5 or 6.5 mg/kg i.v. + pemetrexed 500 mg/m^2^ i.v. + cisplatin 75 mg/m^2^ i.v. Q3W. **safety (*n* = 25):** with pemetrexed + cisplatin, AR MTD was determined to be 6.5 mg/kg. 2 grade 3 AEs occurred (corneal microcystic and hypertension) **efficacy (*n* = 17):** OR (*n* = 17): PR 47% and NE 6%. OR with AR 6.5 mg/kg (*n* = 13): PR 46%	Hassan et al.^199^
		NCT02696642	I	2019-08-19	54	Bayer	evaluation of safety, tolerability, pharmacokinetics, and efficacy of AR	solid	**posology:** AR 6.5 mg/kg i.v. Q3W **safety:** N/A **efficacy:** N/A	–
		NCT02824042	I	2019-08-05	63	Bayer	evaluation of safety, tolerability, pharmacokinetics, and efficacy of AR alone and in combination with itraconazole	solid	**posology:** AR i.v. + atezolizumab i.v. on day 1 (3-week cycles), w/ or w/o itraconazole 200 mg po. twice daily on day 18, once daily on days 19–21, and once daily on days 22–30 **safety:** N/A **efficacy:** N/A	–
Antibody-drug conjugate	Anetumab ravtansine (BAY 94–9343)	NCT01439152	I	2019-07-30	148	Bayer	evaluation of safety, tolerability, pharmacokinetics, and efficacy of AR	solid	**posology:** AR 0.15 to 7.5 mg/kg Q3W. or AR 1.8 or 2.2 mg/kg QW **safety (*n* = 148):** Q3W MTD was determined to be 6.5 mg/kg (*n* = 45), 39% of patients experienced grade ≥3 TESAEs (*n* = 38). QW MTD was determined to be 2.2 mg/kg (*n* = 71), 42% of patients experienced grade ≥3 TESAEs (*n* = 36) **efficacy (*n* = 148):** PFS (*n* = 103): 2.8 months. PFS AR 6.5 mg/kg Q3W (mesothelioma-ovarian, *n* = 12–20): 14.1–4.1 months. PFS AR 2.2 mg/kg QW (mesothelioma-ovarian, *n* = 15–21): 2.6–2.2 months. OR (*n* = 148): CR 1%, PR 7%, SD 45%, PD 41% and NE 7%	Hassan et al.^136^
		NCT02485119	I	2017-07-04	12	Bayer	evaluation of safety, tolerability, pharmacokinetics, and efficacy of AR	solid	**posology:** AR 4.5 or 6.5 mg/kg i.v. Q3W **safety:** N/A **efficacy:** N/A	–
	DMOT4039A	NCT01469793	I	2015-12-01	71	Genentech - Roche	evaluation of safety, tolerability, pharmacokinetics, and efficacy of DMOT4039A	pancreatic and ovarian	**posology:** DMOT4039A 0.2 to 2.8 mg/kg i.v. Q3W, or 0.8 to 1.2 mg/kg i.v. QW **safety:** Most frequent TRAEs occurred in 10% of patients on Q3W and QW schedules. RP2D was determined to be 2.4 mg/kg for Q3W regimen and 1 mg/kg for QW regimen **efficacy pancreatic cancer DMOT4039A 2.4 mg/kg Q3W. (*n* = 26):** PFS: 1.7 months. OR: CR 0%, PR 8%, SD 35%, and PD 57% **efficacy ovarian cancer DMOT4039A 2.4 mg/kg Q3W. (*n* = 10):** PFS: 4.9 months. OR: CR 0% and PR 30%	Weekes et al.^143^
		NCT01832116	I	2014-05-01	11	University Medical Center Groningen	evaluation of pharmacokinetics of 89Zr-MMOT0530A before the clinical trial NCT01469793	pancreatic and ovarian	**posology:** N/A **safety:** N/A **efficacy:** N/A	ter Weele et al.^200^
	BMS-986148	NCT02341625∗	I/II	2020-05-07 (terminated due to business reasons)	126	Bristol-Myers Squibb	evaluation of safety, tolerability, pharmacokinetics, and efficacy of BMS-986148 alone and in combination with nivolumab	solid	**posology:** monotherapy, BMS-986148 0.1 to 1.6 mg/kg i.v. Q3W, or BMS-986148 0.4 to 0.6 mg/kg i.v. QW. In combination, BMS-986148 0.8 mg/kg i.v. Q3W + nivolumab 360 mg i.v. Q3W **safety (*n* = 126):** BMS-986148 MTD and RP2D was determined to be 1.2 mg/kg Q3W (*n* = 84). The dose level of 0.6 mg/kg QW was considered above the MTD (*n* = 12). 49% of patients treated with BMS-986148 monotherapy Q3W experienced grade ≥3 TRAEs (*n* = 84). 25% of patients treated with BMS-986148 monotherapy QW experienced grade ≥3 TRAEs (*n* = 12). 33% of patients treated with BMS-986148 + nivolumab Q3W experienced grade ≥3 TRAEs (*n* = 30) **efficacy BMS-986148 alone 1.2 mg/kg Q3W. (*n* = 51):** PFS (*n* = 51): 2.8 months. PFS (mesothelioma, *n* = 25): 3.8 months. OR (*n* = 44): CR 0%, PR 7%, SD 57%, and PD 36% **efficacy BMS-986148 0.8 mg/kg + nivolumab 360 mg Q3W. (*n* = 30):** PFS (*n* = 30): N/A. PFS (mesothelioma, *n* = 13): 6.5 months. OR (*n* = 26): CR 0%, PR 23%, SD 54%, and PD 23%	Rottey et al.^145^
		NCT02884726	I	2017-09-06	8	Bristol-Myers Squibb	evaluation of safety and tolerability of BMS-986148	N/A	**posology:** BMS-986148 0.8 mg/kg i.v. Q3W **safety (*n* = 7):** 29% of patients experienced grade 3 AEs. No grade 4 AEs or SAEs were reported. One DLT was reported **efficacy:** N/A	Rottey et al.^145^
Antibody-drug conjugate	RC88	NCT06173037	II	estimated 2026-12-31	88	RemeGen	evaluation of safety, pharmacokinetics, and efficacy of RC88	EOC, fallopian, and peritoneal	**posology:** RC88 2 mg/kg i.v. Q3W **safety:** N/A **efficacy:** N/A	–
		NCT06016062	I/II	estimated 2025-12-31	221	RemeGen	evaluation of safety, tolerability, pharmacokinetics, and efficacy of RC148 alone and in combination with docetaxel, RC48, RC88 or RC88 with bevacizumab	solid	**posology:** RC88 2 mg/kg i.v. Q3W + RC148 20 mg/kg i.v. Q3W, w/ or w/o bevacizumab 15 mg/kg i.v. Q3W. (For docetaxel and RC48 details: NCT06016062) **safety:** N/A **efficacy:** N/A	–
		NCT05804526	I/II	estimated 2025-12-01	82	RemeGen	evaluation of safety, pharmacokinetics, and efficacy of RC88 in combination with sintilimab	solid	**posology:** RC88 1.5 to 2.5 mg/kg i.v. Q3W + sintilimab 200 mg i.v. Q3W **safety:** N/A **efficacy:** N/A	–
	RC88	NCT04175847	I/II	estimated 2025-12-01	200	RemeGen	evaluation of safety, tolerability, pharmacokinetics, and efficacy of RC88	solid	**Data cutoff date: 2023-12-19** **posology:** RC88 0.1–2.5 mg/kg i.v. Q3W **safety (*n* = 164):** 40% of patients experienced grade ≥3 TEAEs. 14% of patients experienced TRSAEs. RP2D was determined to be 2 mg/kg Q3W **efficacy (*n* = 6–43):** OR: CR 0%–7% and PR 22%–46%	Liu et al.^147^
		NCT05508334	I	estimated 2025-12-30	81	RemeGen	evaluation of safety, tolerability, and efficacy of RC88	solid	**posology:** RC88 dose escalation i.v. Q2W **safety:** N/A **efficacy:** N/A	Li et al.^146^
	SGN-MesoC2 (PF-08052666 or HBM9033)	NCT06466187	I	estimated 2029-02-16	365	Seagen - Pfizer	evaluation of safety, tolerability, pharmacokinetics, and efficacy of SGN-MesoC2	solid	**posology:** SGN-MesoC2 dose escalation i.v. **safety:** N/A **efficacy:** N/A	Cornelison et al.^148^
Radionuclide therapy	BAY 2287411	NCT03507452	I	2022-03-29	36	Bayer	evaluation of safety, tolerability, pharmacokinetics, and efficacy of BAY2287411	mesothelioma and ovarian	**posology:** Thorium-227 starts at 1.5 MBq and increased in steps of 1.0–1.5 MBq, with antibody doses of 10–400 mg i.v. Q6W **safety:** N/A **efficacy:** N/A	Roy et al.^201^
ADCC and ADCP mediator	Amatuximab (MORAb-009)	NCT02357147∗	II	2018-11-30 (terminated due to business reason)	124	Morphotek	evaluation of safety and efficacy of amatuximab in combination with pemetrexed and cisplatin	MPM	**posology:** amatuximab 5 mg/kg i.v. + pemetrexed 500 mg/m^2^ i.v. + cisplatin 75 mg/m^2^ i.v. Q3W for 6 cycles **safety (*n* = 106):** 20% of patients treated with placebo + pemetrexed + cisplatin experienced SAEs (*n* = 54). 29% of patients treated with amatuximab + pemetrexed + cisplatin experienced SAEs (*n* = 52) **efficacy:** N/A	Hassan et al.^202^
		NCT00738582	II	2014-01-10	89	Morphotek	evaluation of safety, tolerability, and efficacy of amatuximab in combination with pemetrexed and cisplatin	MPM	**posology:** amatuximab 5 mg/kg i.v. on days 1 and 8 + pemetrexed 500 mg/m^2^ i.v. on day 1 + cisplatin 75 mg/m^2^ i.v. on day 1 of each 3-week cycle for 5 cycles **safety (*n* = 89):** 100% of patients experienced TEAEs. 43% of patients experienced SAEs **efficacy (*n* = 89):** OS (*n* = 89): 14.8 months. PFS (*n* = 84): 6.1 months. OR (*n* = 83): CR 0%, PR 40%, SD 51%, and PD 10%	Hassan et al.^153^
		NCT00570713	II	2009-12-01	155	Morphotek	evaluation of safety and efficacy of amatuximab in combination with gemcitabine	pancreatic	**posology:** amatuximab 5 mg/kg i.v. + gemcitabine 200 mg/m^2^ i.v. QW **safety:** 72% of patients treated with placebo + gemcitabine experienced SAEs (*n* = 75). 67% of patients treated with amatuximab + gemcitabine experienced SAEs (*n* = 73) **efficacy placebo + gemcitabine (*n* = 77):** OS: 6.9 months. PFS: 3.5 months. OR: CR 0%, PR 8%, SD 56%, PD 23%, and NE 13% **efficacy amatuximab + gemcitabine (*n* = 78):** OS: 6.5 months. PFS: 3.4 months. OR: CR 0%, PR 6%, SD 47%, PD 19%, and NE 27%	Hassan et al.^152^
		NCT01413451	I	2013-11-15	7	National Cancer Institute	evaluation of safety, tolerability, and pharmacokinetics of ^111^Indium-radiolabeled amatuximab	mesothelioma and PDAC	**posology:** single-dose injection **safety (*n* = 6):** radiotracer dose of 5 mCi was well tolerated **efficacy (*n* = 6):** tumor and metastatic sites imaged. Higher uptake in mesothelioma than pancreatic cancer	Lindenberg et al.^156^
		NCT01521325	I	2013-03-01	6	Morphotek	evaluation of safety, tolerability, and pharmacokinetics of ^111^Indium-radiolabeled amatuximab	mesothelioma and PDAC	**posology:** single-dose injection. **safety (*n* = 6):** radiotracer dose of 5 mCi was well tolerated. **efficacy (*n* = 6):** tumor and metastatic sites imaged. Higher uptake in mesothelioma than pancreatic cancer	Lindenberg et al.^156^
		NCT01018784	I	2013-02-01	17	Eisai	evaluation of safety and tolerability of amatuximab	solid	**posology:** amatuximab 50 to 200 mg/m^2^ i.v. QW **safety (*n* = 17):** MTD was determined to be 200 mg/m^2^. 77% of patients experienced AEs **efficacy (*n* = 17):** OR: CR 0%, PR 0%, SD 18%, and PD 82%	Fujisaka et al.^151^
		NCT00325494	I	2008-09-01	24	Morphotek	evaluation of safety, tolerability, and pharmacokinetics of amatuximab	mesothelioma, pancreatic and ovarian	**posology:** amatuximab 12.5 to 400 mg/m^2^ i.v. on days 1, 8, 15, and 22. **safety (*n* = 24):** MTD was determined to be 200 mg/m^2^.41% patients experienced SAEs, among them 2 patients treated at 400 mg/m^2^ experienced DLT **efficacy (*n* = 24):** OS: 6.5 months. PFS: 3.4 months. OR: CR 0%, PR 0%, SD 46%, PD 38%, and NE 17%.	Hassan et al.^152^
	NI-1801	NCT05403554	I	estimated 2026-09-30	70	Light Chain Bioscience - NovImmune	evaluation of safety, tolerability, pharmacokinetics and efficacy of NI-1801 alone and in combination with pembrolizumab or paclitaxel	ovarian, TNBC, NSCLC, and PDAC	**Data cutoff date: 2025-08-01** **posology:** NI-1801 Q2W or Q3W, w/ or w/o pembrolizumab 400 mg Q6W (up to 4 cycles) or paclitaxel 80 mg/m^2^ QW (up to 4 cycles) **safety (*n* = 42):** well tolerated both as monotherapy and in combination with pembrolizumab **efficacy NI-1801 alone (*n* = 21):** OS (1 year): 75% **efficacy NI-1801 + pembrolizumab (*n* = 21):** OS (1 year): 35%. PFS (9 months): 33%	–
Immunostimulator	ABBV-428	NCT02955251	I	2019-10-29	61	AbbVie	evaluation of safety, tolerability, pharmacokinetics and efficacy of ABBV-428	Solid	**posology:** ABBV-428 0.01 to 3.6 mg/kg i.v. Q2W **safety (*n* = 59):** 3 grade ≥3 AEs were reported. RP2D was determined to be 3.6 mg/kg **efficacy at 3.6 mg/kg (*n* = 25):** OR: CR 0%, PR 0%, SD 36%, and PD 64%	Luke et al.^159^
	AMG 994 (Inezetamab)	NCT04727554	I	2023-06-05	11	Amgen	evaluation of safety, tolerability, pharmacokinetics and efficacy of AMG 994 alone and in combination with AMG 404 (anti-PD-1 IgG1)	Solid	**posology:** AMG 994 (low or high dose) i.v. QW + AMG 404 i.v. Q4W. for 12 cycles (4-week cycles) **safety (*n* = 11):** with low-dose regimen, 0% of patients experienced DLTs and 100% experienced SAEs (*n* = 3). With high-dose regimen, 38% of patients experienced DLTs and 75% experienced SAEs (*n* = 8). 100% of patients experienced TEAEs (*n* = 11) **efficacy low dose (*n* = 3):** OS: 6.0 months. PFS: 2.6 months. OR: CR 0% and PR 0% **efficacy high dose (*n* = 8):** OS: 12.5 months. PFS: 1.6 months. OR: CR 0% and PR 25%	–
T cell engager	HPN536	NCT03872206	I	2023-01-04	95	Harpoon Therapeutics - Merck	evaluation of safety, tolerability, pharmacokinetics, and efficacy of HPN536	N/A	**posology:** HPN536 multiple injection doses of 6–14,400 ng/kg i.v. **safety:** N/A **efficacy:** N/A	Molloy et al.^160^
	AMG 305	NCT05800964	I	estimated 2028-08-12	220	Amgen	evaluation of safety and tolerability of AMG 305	Solid	**posology:** i.v. **safety:** N/A **efficacy:** N/A	Pham et al.^162^
	JNJ-79032421	NCT06255665	I	estimated 2028-03-07	120	Janssen - Johnson & Johnson	evaluation of safety and tolerability of JNJ-79032421	Solid	**posology:** N/A **safety:** N/A **efficacy:** N/A	Smans et al.^163^
	ZW171	NCT06523803∗	I	2025-10-01 (terminated due to sponsor decision)	160	Zymeworks	evaluation of safety, tolerability, and efficacy of ZW171	N/A	**posology:** N/A **safety:** N/A **efficacy:** N/A	–
	CT-95	NCT06756035	I	estimated 2028-12-01	50	Context Therapeutics	evaluation of safety, tolerability, and efficacy of CT-95	solid	**posology:** CT-95 dose escalation i.v. QW. **safety:** N/A **efficacy:** N/A	–

Discontinued clinical trials are shown with the symbol ∗. ADCC, antibody-dependent-cell-mediated cytotoxicity; ADCP, antibody-dependent-cell-mediated phagocytosis; AE, adverse event; AR, anetumab ravtansine; CHO, cholangiocarcinoma; CR, complete response; DLT, dose-limiting toxicity; EOC, epithelial ovarian cancer; FDA, Food and Drug Administration; HGOC, high-grade ovarian cancer; itu., intratumoral; i.v., intravenous; MPM, malignant pleural mesothelioma; MTD, maximum tolerated dose; NCI, National Cancer Institute; NE, not evaluated; NSCLC, non-small-cell lung cancer; OR, overall response; OS, overall survival; PD, progressive disease; PDAC, pancreatic ductal adenocarcinoma; PFS, progression-free survival; PLD, pegylated liposomal doxorubicin; po., per os; PR, partial response; QW, once weekly; Q2W, once every 2 weeks; Q3W, once every 3 weeks; Q6W, once every 6 weeks; QOD, every other day; RP2D, recommended phase 2 dose; SAE, serious adverse event; SD, stable disease; TEAE, treatment emergent adverse event; TESAE, treatment emergent serious adverse event; TNBC, triple negative breast cancer; TRAE, treatment-related adverse event; TRSAE, treatment-related serious adverse event; TTP, time to progression.

To overcome the immunogenicity issues, the research shifted to a second generation of iTOX with LMB-100. LMB-100 is a humanized anti-MSLN Fab fused to the deimmunized domain III of *Pseudomonas* exotoxin A (24 kDa) to reduce immunogenicity.^126^ Nine clinical trials with LMB-100 alone or in combination with chemotherapy or ICIs have been reported, most of them in MPM (Table 2). LMB-100 alone was found to be well tolerated with manageable side effects.^131^^,^^132^ However, despite a lower immunogenicity than SS1P, it still faces the formation of ADA, limiting its long-term use in patients.^133^ According to the latest update from AdisInsight, its development has been discontinued for solid tumors.

Interestingly, although most epithelioid MPMs express MUC16, the impact of its presence on the therapeutic efficiency of iTOX that compete with MUC16 has not been thoroughly investigated.

As of March 2025, there is no evidence of ongoing clinical trials with SS1P or LMB-100 (Figure 4B).

#### ADCs: Anetumab ravtansine, DMOT4039A, BMS-986148, RC88, and SGN-MesoC2

Antibody-drug conjugates (ADCs) combine the specificity of monoclonal antibodies with the potency of cytotoxic payloads.^134^

Anetumab ravtansine (BAY 94–9343 or AR) is an ADC developed by Bayer. It is a human anti-mesothelin antibody that does not compete with MUC16. The antibody is conjugated to the maytansinoid tubulin inhibitor DM4 via a non-cleavable disulfide-containing linker (Figure 4A).^135^ To date, 15 clinical trials have been conducted with AR, the first of which was initiated in 2011 (Table 2).^136^ AR has been evaluated both as monotherapy and in combination with immune checkpoint inhibitors (ICIs) (atezolizumab, bevacizumab, ipilimumab, nivolumab, and pembrolizumab), chemotherapy (cisplatin, gemcitabine, pegylated liposomal doxorubicin [PLD], and pemetrexed), or fungicide (itraconazole). Regarding safety, clinical studies have demonstrated that AR exhibits a satisfactory level of tolerability and favorable pharmacokinetics across multiple studies. Concerning its efficacy, there is evidence supporting the clinical potential of AR in some MSLN-expressing tumors, with cases of complete responses (CRs) and partial responses (PRs) reported.^136^^,^^137^ However, a direct comparison with other treatments, such as vinorelbine, has not yet demonstrated the superiority of AR in terms of progression-free survival (PFS) or overall survival (OS).^136^^,^^137^ While it has shown promise as part of combination therapies in certain contexts, particularly in high mesothelin-expressing tumors, its clinical efficacy remains inconsistent across indications.^138^^,^^139^^,^^140^ AR is still in an active phase 2 clinical trial, notably in combination with ICI.^138^

DMOT4039A is an ADC developed by Genentech, a subsidiary of Roche. The structure of DMOT4039A consists of a humanized anti-MSLN monoclonal antibody (mAb) (h7D9.v3 or MMOT0530A), which does not compete with MUC16 binding conjugated to the tubulin inhibitor monomethyl auristatin E (MMAE) via a cathepsin-cleavable linker (Figure 4A).^141^^,^^142^ To date, two related phase 1 clinical trials have been conducted with DMOT4039A as monotherapy, focusing on pancreatic and ovarian cancers (Table 2). The first study, initiated in 2011, demonstrated acceptable tolerability but yielded disappointing results regarding clinical activity, particularly in patients with pancreatic cancer.^143^ A confirmed partial response was observed in only 8% of patients, compared to 30% in ovarian cancer patients.^143^ The second one, a multicenter imaging study (NCT01832116) performed in parallel using the same antibody conjugated to zirconium-89 showed a lower uptake in the pancreatic tumors than in ovarian tumors, which may be due to the high level of fibrosis in the pancreatic microenvironment.^144^ Since 2015, the development status of DMOT4039A remains unknown.

The ADC BMS-986148, a product of Bristol-Myers Squibb, is a fully human anti-MSLN IgG1 conjugated to a cytotoxic peptide, tubulysin, a tubulin polymerization inhibitor via a cleavable linker (Figure 4A).^145^ There is no information on the epitope targeted by BMS-986148. BMS-986148 has been evaluated both in monotherapy and in combination with nivolumab in different indications (Table 2). The phase 1 assay as monotherapy in advanced and/or metastatic solid tumors was completed in 2017, and the second trial was prematurely terminated. In both trials, BMS-986148 demonstrated a manageable safety profile in patients with advanced tumors and a durable antitumor activity was observed in some patients. Notably, treatment discontinuations were observed in both assays, primarily due to treatment-related adverse events (TRAEs).^145^ The status of any ongoing research by Bristol-Myers Squibb on this compound remains uncertain.

RC88, developed by RemeGen, consists of an anti-mesothelin antibody linked to MMAE via a cleavable linker (Figure 4A).^146^ No information regarding the targeted epitope on MSLN has been disclosed. At present, five clinical trials are underway with RC88, either as monotherapy or in combination with monoclonal antibodies (mAbs), including RC48 (anti-HER2), bevacizumab (anti-VEGF), and sintilimab (anti-PD1) (Table 2). To date, there have been no published clinical trial data, apart from a poster presented at the 2024 ASCO meeting that reported the tolerability and clinical efficacy of RC88 in patients with mesothelin-expressing solid tumors.^147^ Following the FDA’s approval of the fast track designation for RC88 in December 2023, a phase 2 clinical trial has been initiated to assess the efficacy of RC88 in patients diagnosed with platinum-resistant recurrent epithelial ovarian, fallopian tube, and primary peritoneal cancer.

SGN-MesoC2 (or PF-08052666) is an ADC initially developed by Harbour BioMed under the name HBM9033 but now licensed by Pfizer. SGN-MesoC2, described as binding to membrane-bound MSLN, comprises a camptothecin-based topoisomerase 1 inhibitor payload (SGD-12280) coupled to a human immunoglobulin G1 (IgG1) mAb via a protease-cleavable linker (Figure 4A).^148^ SGN-MesoC2 is the only anti-MSLN ADC in clinical trials that does not use a microtubule inhibitor as payload.^134^ The first clinical trial started in 2024.

#### TRT: BAY 2287411

BAY 2287411 is a mesothelin-targeted thorium-227 conjugate developed by Bayer. The human anti-mesothelin antibody (BAY 86–1903) is conjugated to the 3,2-HOPO chelator and radiolabeled with the alpha emitter thorium-227 (Figure 4A).^149^ To date, only one clinical trial has been conducted with BAY 2287411: a phase 1 monotherapy study that began in 2018 and ended in 2022 (Table 2). The results of this study are not available. Preclinical studies have demonstrated that BAY 2287411 is well tolerated in mice and in monkeys, induces a specific and significant antitumor activity, and activates several immunostimulatory pathways while modulating the surface expression of various immunomodulatory markers.^149^ Furthermore, the combination of BAY 2287411 with either ICIs or DNA damage response inhibitors led to a significant potentiation of the antitumor activity of the compound.^149^^,^^150^

#### ADCC and ADCP mediators: Amatuximab and NI-1801

Amatuximab (or Morab-009), developed by Morphotek, is a humanized chimeric IgG1 derived from SS1, therefore competing with MUC16 (Figure 4A). Its mechanism of action includes inhibition of the MSLN/MUC16 axis and ADCC/ADCP. Six clinical trials have been conducted with amatuximab alone or in combination with pemetrexed and cisplatin (Table 2), in MPM and PDAC. However, despite a good safety profile, clinical studies concluded that amatuximab cannot be regarded as an effective treatment.^151^^,^^152^ Interestingly, based on the clinical trial NCT00738582, which did not achieve its targeted PFS primary endpoint, Nicolaides et al. investigate the correlation between MUC16 expression levels and the efficacy of amatuximab-based treatments.^153^^,^^154^ The authors demonstrated that amatuximab treatment induced a reversible increase in serum CA-125 levels due to the blockade of MUC16-MSLN interactions. Furthermore, soluble CA-125 was shown to impair amatuximab-mediated immune effector functions by binding to its (Fab’)_2_ domain, thereby disrupting its interactions with CD16a (ADCC) and CD32a (ADCP) receptors.^154^^,^^155^ No major studies have been published since the discontinued trial NCT02357147.

Amatuximab has also been investigated as an imaging agent. Two correlated phase 1 clinical trials were conducted with ^111^Indium-radiolabeled amatuximab in mesothelioma and PDAC patients.^156^ The efficacy of the agent in imaging tumor and metastatic sites was demonstrated, thus supporting its potential application as a diagnostic tool.

NI-1801, developed by Light Chain Bioscience-NovImmune, is a bispecific human IgG1 κλ-body targeting mesothelin and CD47 on tumors cells (Figure 4A). As described by the company, NI-1801 is made of a common human IgG1 heavy chain and two light chains (one kappa and one lambda) with distinct specificities. NI-1801 relies on ADCC and ADCP mechanisms, the ADCP process being enhanced by the blocking of the CD47 “don’t eat me signal.” Based on the publication of Hatterer et al., it is likely that the MSLN arm targets a membrane-proximal epitope on MSLN, thus not competing with MUC16.^157^ A phase 1 study has started in 2022 with solid tumors.

#### Immunostimulators: ABBV-428 and AMG 994

ABBV-428 and AMG 994, developed by AbbVie and Amgen, respectively, are bispecific antibodies that exhibit bivalency toward mesothelin and CD40. ABBV-428 is an scFv-based antibody composed of a silent human IgG1 Fc fused to anti-CD40 and anti-MSLN scFv at the N- and C-terminus, respectively (Figure 4A).^158^ As described in the Amgen protocol 20190136, AMG 994, also known as inezetamab, is an effector-silenced anti-CD40 IgG1 fused to an anti-MSLN scFv at the C-terminus. There is no information about the targeted epitope on MSLN. The mechanism of action of both molecules relies on the MSLN-dependent activation of CD40-expressing immune cells, leading to an enhanced priming and activation of cytotoxic T cells, activation of B lymphocytes, and reprogramming of type 2 macrophages into type 1.^158^^,^^159^ Phase 1 clinical trials have been conducted to assess the efficacy of both molecules in MSLN-positive solid tumors (Table 2). ABBV-428 evaluated as monotherapy demonstrated acceptable tolerability, but only minimal clinical activity. No further development of this molecule has been reported. Preliminary results suggest that AMG 994 demonstrates higher efficacy, yet it concomitantly induces toxicity. However, the development of AMG 994 was discontinued following a business decision.

#### T cell engagers: HPN536, AMG 305, JNJ-79032421, ZW171, and CT-95

HPN536, AMG 305, JNJ-79032421, ZW171, and CT-95 are all anti-MSLN/anti-CD3 TCEs able to recruit and activate T cells against MSLN^+^ tumor cells (Figure 4A).

HPN536 is a trispecific T-cell-activating construct (TriTAC) developed by Harpoon Therapeutics, a subsidiary of Merck.^160^ It consists of a fusion of a humanized anti-MSLN llama single-domain antibody (sdAb), an anti-human serum albumin (HSA) sdAb for half-life extension through the FcRn loop, and a humanized scFv anti-CD3ε.^161^ The building blocks are connected by G_4_SG_3_S linkers.^160^

AMG 305, a trispecific antibody-like TCE directed against MSLN (monovalent), p-cadherin (CDH3) (monovalent), and CD3 (bivalent), was developed by Amgen. This agent is an scFv-based construct that requires binding to both CDH3 and MSLN for potent cytotoxic activity, CDH3 and MSLN being co-expressed in multiple solid tumor types.^162^

JNJ-79032421 is a membrane-restricted mesothelin targeting Fab fused to an anti-CD3 scFv developed by Janssen, a Johnson & Johnson subsidiary.^163^ The precise structure of the compound remains undisclosed.

ZW171 is a 2 + 1 scFv-Fab-based anti-MSLN (bivalent) and anti-CD3 (monovalent) TCE, developed by Zymeworks.^164^ The only clinical trial (NCT06523803) conducted with this therapeutic agent was discontinued, likely due to on-target/off-tumor toxicity.

CT-95 is a 2 + 2 bispecific scFv-Fab-based TCE anti-MSLN (bivalent) and anti-CD3 (bivalent) developed by Context Therapeutics. As described by the company, it has been engineered to target a membrane proximal epitope on MSLN.

In cynomolgus monkeys, HPN536 demonstrated both good tolerability and MSLN-dependent pharmacologic activity.^160^ In cynomolgus monkeys, AMG 305 and ZW171 also demonstrated good tolerability.^162^ In a CDH3^+^ MSLN^+^ xenograft tumor model, AMG 305 has been shown to demonstrate *in vivo* antitumor activity that is dose dependent.^162^

To date, HPN536, AMG 305, JNJ-79032421, ZW171, and CT-95 are all involved in only one monotherapy phase 1 study. The trials were initiated in 2019, 2023, 2024, 2024, and 2025, respectively. The results of these studies are not yet available (Table 2).

### Adoptive cell therapies targeting mesothelin

Mesothelin-targeted cell therapies have made notable progress in recent years, with several approaches progressing toward early-phase clinical trials. While the majority of adoptive cell therapies targeting mesothelin are based on conventional αβ T cell engineering, in particular chimeric antigen receptor (CAR)-T cells, the field is actively exploring other immune cell types (such as NK cells, γδ T cells, or macrophages) for CAR-based therapies, especially in the context of solid tumors.

#### MSLN-targeting T cells

The clinical research on anti-MSLN CAR-T cells was initiated in 2011 with the mRNA-transfected SS1/CD3ζ/4-1BB CAR-T cells developed by the University of Pennsylvania.^165^ Fifteen years later, various strategies employing CAR-T cells have advanced to the clinical stage with currently 63 clinical trials registered (Table 3).

**Table 3:** MSLN-targeted cell therapies in clinical trials

Cell type	Drug name	NCT number	Phase	Completion date	Enrollment	Sponsor	Study	Tumor type	Study outcomes	Reference
CAR-T cells	anti-MSLN CAR-T cells (mRNA transfected SS1/CD3ζ/4-1BB CAR-T cells)	NCT01897415	I	2017-03-01	16	University of Pennsylvania	evaluation of feasibility, safety, and efficacy of anti-MSLN CAR-T cells	PDAC	**posology:** no LD. 1 to 3 × 10^8^ cells/m^2^ i.v. 3 times weekly for 3 weeks. **safety (*n* = 6):** no DLT reported. No CRS reported. 3 grade 3 AEs were reported. 2 grade 4 TRAEs were reported. **efficacy (*n* = 6):** PFS: 4.6 months. OR: CR 0%, PR 0%, SD 33%, and PD 67%.	Beatty et al.^166^
		NCT01355965	I	2015-10-01	18	University of Pennsylvania	evaluation of feasibility, safety, pharmacokinetics, and efficacy of anti-MSLN CAR-T cells	MPM and PDAC	**Data cutoff date: 2014-02-01** **efficacy:** no LD. Different protocols were used with multiple injections at 1 × 10^8^ cells i.v., 1 × 10^9^ cells i.v., 3 × 10^8^ cells/m^2^ i.v. and/or 2 × 10^8^ cells itu. **safety (*n* = 2):** treatment not tolerated, the first patient died because of the treatment and the second experienced 2 grade ≥3 AEs **efficacy (*n* = 2):** results suggest a role of anti-MSLN CAR-T cells in inducing an antitumor effect	Beatty et al.^165^
	anti-MSLN CAR-T cells (retroviral transduced CAR-T cells)	NCT01583686∗	I/II	2018-12-17 (terminated due to slow/insufficient accrual)	15	National Cancer Institute	evaluation of safety, tolerability, and efficacy of anti-MSLN CAR-T cells in combination with aldesleukin	solid	**posology:** LD. Single-dose injection of 1 × 10^6^ to 1 × 10^8^ cells i.v. + aldesleukin 72,000 IU/kg i.v. (up to 15 dose injections) **safety (*n* = 15):** 45% of patients experienced SAEs. The only group without SAEs is the one with 1 × 10^7^ cells **efficacy (*n* = 15):** OR: CR 0%, PR 0%, SD 7%, and PD 93%. The only patient with an SD was injected with 3 × 10^6^ CAR-PBL	–
	huCART-meso cells(lentiviral transduced SS1/4-1BB/CD3ζ CAR-T cells)	NCT05057715	I	estimated 2038-09-01	13	University of Pennsylvania	evaluation of feasibility and safety of huCART-meso cells in combination with VCN-01	pancreatic and ovarian	**posology:** No LD. VCN-01 3.3 × 10^12^ vp i.v. on day 0 + huCART-meso 5 × 10^7^ cells i.v. on day 14 (regimen A). VCN-01 1 × 10^13^ vp i.v. on day 0 + huCART-meso 5 × 10^7^ cells i.v. on day 14 (regimen B). huCART-meso cells i.v. on day 0 + VCN-01 3.3 × 10^12^ vp i.v. on day 14 (regimen C) **safety:** N/A **efficacy:** N/A	–
		NCT05623488	I	estimated 2038-02-01	12	University of Pennsylvania	evaluation of feasibility and safety of intratumoral huCART-meso cells injection	TNBC	**posology:** no LD. 3 × 10^6^ to 3 × 10^7^ cells itu **safety:** N/A **efficacy:** N/A	–
		NCT03323944	I	estimated 2025-09-01	18	University of Pennsylvania	evaluation of feasibility and safety of i.v. injection and local delivery of huCART-meso cells	pancreatic	**safety:** no LD. Single-dose injection of 1 × 10^6^ to 3 × 10^7^ cells/m^2^ i.v., i.p., or intrahepatic. **safety:** N/A **efficacy:** N/A	Haas et al.^123^
		NCT03054298	I	2024-07-30	54	University of Pennsylvania	evaluation of feasibility and safety of i.v. injection and local delivery of huCART-meso cells	mesothelioma, ovarian, and pancreatic	**Data cutoff date: 2019-07-17** **posology:** w/ or w/o LD. Single-dose injection of 1–3 × 10^7^ cells/m^2^ i.v. or 1 to 3 × 10^8^ cells/m^2^ i.v. **safety (*n* = 15):** 7 grade ≥3 AEs were reported **efficacy (*n* = 15):** PFS: 2.1 months. OR: CR 0%, PR 0%, SD 73%, and PD 27%	Haas et al.^123^
CCAR-T cells	huCART-meso cells (lentiviral transduced SS1/4-1BB/CD3ζ CAR-T cells)	NCT02465983∗	I	2017-11-01 (teminated due to lack of efficacy and funding)	4	University of Pennsylvania	evaluation of feasibility, safety, pharmacokinetics, and efficacy of huCART-meso cells in combination with anti-CD19 CAR-T cells	PDAC	**posology:** LD. huCART-meso cells 3 × 10^7^ cells/m^2^ i.v. + anti-CD19 CAR-T cells 3 × 10^7^ cells/m^2^ i.v. (two separate injections) **safety (*n* = 3):** no DLT reported **efficacy (*n* = 3):** OR: CR 0%, PR 0%, SD 33%, PD 67%. CART-19 cells deplete normal B cells but at the dose tested no improvement of huCART-meso cells persistence	Ko et al.^203^
		NCT02159716	I	2015-11-01	19	University of Pennsylvania	evaluation of feasibility, safety, pharmacokinetics, and efficacy of huCART-meso cells	PDAC, EOC, and MPM	**posology:** w/ or w/o LD. Single-dose injection of 1–3 × 10^7^ cells/m^2^ i.v. or 1 to 3 × 10^8^ cells/m^2^ i.v. **safety (*n* = 15):** without LD, at 1 to 3 × 10^7^ cells/m^2^ a DLT occurred **efficacy (*n* = 15):** PFS: 2.1 months. OR: CR 0%, PR 0%, SD 73%, and PD 27%	Haas et al.^123^
		NCT02388828	N/A	2019-07-01	10	University of Pennsylvania	evaluation of long-term safety of huCART-meso cells	N/A	**posology:** no LD **safety:** N/A **efficacy:** N/A	–
	M28z cells (retroviral transduced anti-MSLN M912 scFv/CD28/CD3ζ CAR-T cells)	NCT02414269	I/II	estimated 2026-04-30	113	Memorial Sloan Kettering Cancer Center	evaluation of safety and efficacy of M28z cells alone and in combination with pembrolizumab	solid	**Data cutoff date: 2019-04-01** **posology:** w/ or w/o LD. Single-dose injection of 1.3 × 10^5^ to 8.6 × 10^7^ cells/kg ip. (at PD stage 15% of patients received a second injection) + pembrolizumab 200 mg i.v. 3 times and 4 weeks after completing CAR-T cells injection **safety (*n* = 27):** no DLTs reported. 22% of patients experienced grade 3 AEs. Grade 4 AEs were reversible **efficacy M28z + pembrolizumab (*n* = 16):** OS: 23.9 months. OR: CR 0%, PR 13%, SD 56%, and PD 31%	Adusumilli et al.^167^
	anti-MSLN CAR-T cells (retroviral transduced anti-MSLN/4-1BB/CD3ζ CAR-T cells)	NCT02580747∗∗	I	estimated 2018-11-01 (unknown status)	20	Chinese PLA General Hospital	evaluation of feasibility, safety, and efficacy of anti-MSLN CAR-T cells	solid	**posology:** no LD. Injection on days 0, 1, and 2 **safety:** N/A **efficacy:** N/A	–
	TAI-meso-CART cells	NCT02706782∗∗	I	estimated 2018-09-01 (unknown status)	30	Shanghai GeneChem	evaluation of safety and efficacy of TAI-meso-CART cells	pancreatic	**posology:** LD. Single-dose injection of 1 × 10^6^ to 10 × 10^6^ cells/kg i.v. **safety:** N/A **efficacy:** N/A	–
	anti-MSLN CAR-T cells (transduced anti-MSLN CAR-T cells)	NCT02792114	I	estimated 2026-06-01	186	Memorial Sloan Kettering Cancer Center	evaluation of safety and tolerability of anti-MSLN CAR-T cells	breast	**posology:** LD. Single-dose injection i.v. **safety:** N/A **efficacy:** N/A	–
	anti-MSLN CAR-T cells (lentiviral transduced CAR-T cells)	NCT02959151∗∗	I/II	estimated 2018-07-01 (unknown status)	20	Shanghai GeneChem	evaluation of safety and efficacy of anti-MSLN CAR-T cells in combination with interventional therapy	pancreatic	**posology:** no LD. Single-dose injection of 1.25–4 × 10^7^ cells/cm^3^ of tumor bulk i.v. or itu **safety:** N/A **efficacy:** N/A	–
	anti-MSLN CAR-T cells	NCT02930993∗∗	I	estimated 2019-08-01 (unknown status)	20	China Meitan General Hospital	evaluation of safety and efficacy of anti-MSLN CAR-T cells	solid	**posology:** LD. 3-day split-dose of 5 × 10^4^ to 1 × 10^7^ cells/kg i.v. (day 0,10%; day 1, 30%; day 2, 60%). **safety:** N/A **efficacy:** N/A	–
	αPD-1-mesoCAR-T cells (DNA transfected anti-MSLN CAR-T cells secreting PD-1 antibody)	NCT03615313∗∗	I/II	estimated 2020-12-03 (unknown status)	50	Shanghai Cell Therapy Research Institute	evaluation of safety and efficacy of αPD-1-mesoCAR-T cells	solid	**Data cutoff date: 2020-05-25** **posology:** LD. 5 × 10^6^ cells/kg, 5 × 10^7^ cells/kg or 1 × 10^8^ cells/kg i.v. from day 1 to day 3 **safety (*n* = 10):** most common AEs were mild fatigue and fever **efficacy (*n* = 10):** PFS: 3.2 months. OR: CR 0%, PR 20%, SD 40%, and PD 40%	Fang et al.^169^
CAR-T cells	αPD-1-mesoCAR-T cells (DNA transfected anti-MSLN CAR-T cells secreting PD-1 antibody)	NCT03030001∗∗	I/II	estimated 2019-02-01 (unknown status)	40	Ningbo Cancer Hospital	evaluation of safety and efficacy of anti-MSLN CAR-T cells expressing PD-1 antibody	solid	**posology:** no LD. Single-dose injection i.v. **safety:** N/A **efficacy:** N/A	–
	anti-MSLN CAR-T cells secreting CTLA-4/PD-1 antibodies	NCT03182803∗∗	I/II	estimated 2019-04-20 (unknown status)	40	Shanghai Cell Therapy Research Institute	evaluation of safety, pharmacokinetics, and efficacy of anti-MSLN CAR-T cells secreting CTLA-4/PD-1 antibodies	solid	**posology:** LD. 2 to 5 × 10^7^ cells/kg i.v. from day 18 to day 19 (±2 days), 2 cycles **safety:** N/A **efficacy:** N/A	–
	anti-MSLN CAR-T cells	NCT03267173∗∗	I	estimated 2019-06-01 (unknown status)	10	First Affiliated Hospital of Harbin Medical University	evaluation of safety and efficacy of anti-MSLN CAR-T cells	pancreatic	**posology:** no LD. Single-dose injection **safety:** N/A **efficacy:** N/A	–
		NCT04203459∗∗	N/A	estimated 2022-12-31 (unknown status)	80	First Affiliated Hospital of Harbin Medical University	evaluation of the effects and mechanism of gut microbiota on anti-MSLN CAR-T cells	pancreatic	**posology:** N/A **safety:** N/A **efficacy:** N/A	–
	anti-MSLN CAR-T cells (anti-MSLN CD8^+^ CAR-T cells + anti-TGF-β CD4^+^ CAR-T cells secreting IL-7/CCL19 and/or anti-PD-1/CTLA-4/TIGIT scFvs)	NCT03198052	I	estimated 2036-08-01	30	Second Affiliated Hospital of Guangzhou Medical University	evaluation of safety, tolerability, and efficacy of anti-MSLN CD8^+^ CAR-T cells + anti-TGF-β CD4^+^ CAR-T cells secreting IL-7/CCL19 and/or anti-PD-1/CTLA-4/TIGIT scFvs	lung	**posology:** no LD. 1 to 10 × 10^6^ cells/kg i.v. or regional injections for 3 or more cycles **safety:** N/A **efficacy:** N/A	–
	anti-MSLN-7 × 19 CAR-T cells (lentiviral transduced anti-MSLN scFv/TLR2/CD3ζ CAR-T cells secreting human IL-7 and CCL19)	NCT03198546	I	estimated 2036-08-01	30	Second Affiliated Hospital of Guangzhou Medical University	evaluation of safety and efficacy of anti-MSLN-7 × 19 CAR-T cells	pancreatic and ovarian	**Data cutoff date: 2021-07-17** **posology:** no LD. Injections every 1 to 2 months i.v. or local **safety (*n* = 6):** injection every 1 to 2 months. No grade ≥2 AEs reported **efficacy (*n* = 2):** OR: CR 50%, PR 0%, SD 0%, and PD 50%	Pang et al.^204^
	anti-MSLN CAR-T cells(lentiviral transduced anti-MSLN scFv/4-1BB/CD3ζ CAR-T cells)	NCT03497819∗∗	I	estimated 2020-10-31 (unknown status)	10	First Affiliated Hospital of Wenzhou Medical University	evaluation of safety and efficacy of anti-MSLN CAR-T cells in combination with anti-CD19 CAR-T cells	pancreatic	**posology:** LD. i.v. or artery injection **safety:** N/A **efficacy:** N/A	–
	anti-MSLN CAR-T cells (GIMI-IRB cells)	NCT03356808∗∗	I/II	estimated 2020-12-31 (unknown status)	20	Shenzhen Geno-Immune Medical Institute	evaluation of feasibility, safety, pharmacokinetics, and efficacy of anti-MSLN CAR-T cells	lung (SCLC and NSCLC)	**posology:** single-dose injection of 1 × 10^6^ to 1 × 10^7^ cells/kg i.v. **safety:** N/A **efficacy:** N/A	–
CAR-T cells	anti-MSLN CAR-T cells (GIMI-IRB cells)	NCT03356795∗∗	I/II	estimated 2020-12-01 (unknown status)	20	Shenzhen Geno-Immune Medical Institute	evaluation of feasibility, safety, pharmacokinetics, and efficacy of anti-MSLN CAR-T cells	cervical	**posology:** no LD. Single-dose injection of 1 × 10^6^ to 1 × 10^7^ cells/kg i.v. **safety:** N/A **efficacy:** N/A	–
	anti-MSLN CAR-T cells	NCT03638206∗∗	I/II	estimated 2023-03-01 (unknown status)	73	Shenzhen BinDeBio	evaluation of safety and efficacy of anti-MSLN CAR-T cells	gastric, pancreatic, and mesothelioma	**posology:** LD. Single-dose injection **safety:** N/A **efficacy:** N/A	–
		NCT03941626∗∗	I/II	estimated 2021-12-01 (unknown status)	50	Shenzhen BinDeBio	evaluation of safety and efficacy of anti-MSLN CAR-T cells	gastric	**posology:** no LD **safety:** N/A **efficacy:** N/A	–
	MPTK-CAR-T cells (PD-1 and TCR deficient lentiviral transduced P4 scFv/CD28/CD3ζ CAR-T cells)	NCT03545815∗∗	I	estimated 2020-12-30 (unknown status)	15	Chinese PLA General Hospital	evaluation of feasibility, safety, pharmacokinetics, and efficacy of MPTK-CAR-T cells	solid	**posology:** no LD. Single-dose injection of 0.1–9 × 10^6^ cells/kg i.v. If undetectable circulating MPTK-CAR-T cells, possibility of a second or a third infusion (8/15) **safety (*n* = 15):** 5 grade ≥3 AEs were reported **efficacy (*n* = 15):** PFS: 1.6 months. OR: CR 0%, PR 0%, SD 13%, and PD 87%	Wang et al.^205^
	GC008t cells ((PD-1 deficient lentiviral transduced P4 scFv/CD28/CD3ζ CAR-T cells))	NCT03747965∗∗	I	estimated 2020-05-01 (unknown status)	10	Chinese PLA General Hospital	evaluate the feasibility, safety, and efficacy of GC008t cells	pancreatic, ovarian, and colorectal	**Data cutoff date: 2020-01-20** **posology:** w/ or w/o LD. 1 × 10^6^ to 5 × 10^7^ cells/kg. 4/9 patients received repeat injections of GC008t **safety (*n* = 9):** CRS noticed only at the highest dose **efficacy (*n* = 9):** PFS (PR condition, *n* = 2): 3.9 months. OR (*n* = 9): CR 0%, PR 22%, SD 44%, PD 11%, and NE 22%	Wang et al.^206^
	anti-MSLN CAR-T cells (lentiviral transduced SS1/TCRζ/4-1BB CAR-T cells)	NCT03638193∗∗	N/A	estimated 2022-02-01 (unknown status)	10	Shenzhen BinDeBio	evaluation of safety and efficacy of CART-meso cells	pancreatic	**posology:** LD. Single-dose injection of 1 × 10^7^ to 3 × 10^8^ cells/m^2^ i.v. **safety:** N/A **efficacy:** N/A	–
	anti-MSLN CAR-T cells (retroviral transduced CAR-T cells)	NCT03799913∗∗	I	estimated 2022-04-01 (unknown status)	20	Zhejiang University	evaluation of feasibility, safety, pharmacokinetics, and efficacy of anti-MSLN CAR-T cells	ovarian	**posology:** LD. **safety:** N/A **efficacy:** N/A	–
	gavo-cel (gavocabtagene autoleucel or TC-210 or lentiviral transduced anti-MSLN sdAb (MH1)/CD3ζ CAR-T cells)	NCT03907852	I/II	estimated 2028-11-02	36	TCR2 Therapeutics	evaluation of safety and efficacy of gavo-cel	MPM, ovarian, and CHO	**Data cutoff date: 2023-01-17** **posology:** w/ or w/o LD. Single-dose injection of 5 × 10^7^ to 5 × 10^8^ cells/m^2^ i.v. **safety (*n* = 32):** 97% of patients experienced AEs. 78% of patients experienced CRS. The RP2D was determined to be 1 × 10^8^ cells/m^2^, after LD. At the RP2D, no cases of pneumonitis were reported and severe CRS events (2/13, 15%) were rapidly reversible **efficacy in MPM (*n* = 23):** OS: 11.1 months. PFS: 5.6 months **efficacy in ovarian cancer (*n* = 8):** OS: 9.4 months. PFS: 5.8 months	Hassan et al.^174^
	anti-MSLN CAR-T cells (retroviral transduced CAR-T cells)	NCT03916679∗∗	I/II	estimated 2023-04-20 (unknown status)	20	Second Affiliated Hospital, School of Medicine, Zhejiang University	evaluation of feasibility, safety, pharmacokinetics, and efficacy of anti-MSLN CAR-T cells	EOC	**posology:** LD. **safety:** N/A **efficacy:** N/A	–
CAR-T cells	anti-MSLN CAR-T cells (transduced anti-MSLN CAR-T cells)	NCT03814447∗∗	I	estimated 2023-01-01 (unknown status)	10	Shanghai 6th People’s Hospital	evaluation of feasibility, safety, pharmacokinetics, and efficacy of anti-MSLN CAR-T cells	Ovarian	**posology:** LD. Single-dose injection of 5 × 10^6^ cells/kg **safety:** N/A **efficacy:** N/A	–
	αPD1-MSLN-CAR T cells (anti-MSLN CAR-T cells secreting anti-PD-1 sdAb)	NCT05089266	I	estimated 2025-01-30	30	Shanghai Cell Therapy Group	evaluation of safety and efficacy of αPD1-MSLN-CAR T cells	Colorectal	**posology:** No LD. **safety:** N/A **efficacy:** N/A	–
		NCT05373147∗∗	I	estimated 2023-08-01 (unknown status)	21	Shanghai Mengchao Cancer Hospital	evaluation of safety, tolerability, pharmacokinetics and efficacy of αPD1-MSLN-CAR T cells	solid	**posology:** no LD. Single-dose injection of 1 × 10^5^ to 3 × 10^6^ cells/kg **safety:** N/A **efficacy:** N/A	–
		NCT04489862∗∗	I	estimated 2022-12-01 (unknown status)	10	Wuhan Union Hospital, China	evaluation of safety, tolerability, pharmacokinetics and efficacy of αPD1-MSLN-CAR T cells	NSCLC and mesothelioma	**posology:** LD. Single-dose injection of 1 × 10^5^ to 3 × 10^6^ cells/kg i.v. **safety:** N/A **efficacy:** N/A	–
		NCT04503980∗∗	I	estimated 2022-06-01 (unknown status)	10	Shanghai Cell Therapy Group	evaluation of safety, tolerability, pharmacokinetics, and efficacy of αPD1-MSLN-CAR T cells	colorectal and ovarian	**safety:** LD. 1 × 10^5^ to 3 × 10^6^ cells/kg i.v. **safety:** N/A **efficacy:** N/A	–
	M28z1XXPD1DNR cells (M28z equipped with a modified CD3ζ (1XX), and a PD-1 dominant negative receptor (PD1DNR))	NCT06623396	I	estimated 2028-09-30	18	Memorial Sloan Kettering Cancer Center	evaluation of safety and tolerability of M28z1XXPD1DNR cells	esophagogastric	**posology:** no LD. Single-dose injection i.p. **safety:** N/A **efficacy:** N/A	–
		NCT04577326	I	estimated 2026-09-30	14	Memorial Sloan Kettering Cancer Center	evaluation of safety and pharmacokinetics of M28z1XXPD1DNR cells	MPM	**Data cutoff date: 2021-12-01** **posology:** LD. Single-dose injection of 1–3 × 10^6^ cells/kg ip. **safety (*n* = 4):** No grade ≥3 TRAEs reported. **efficacy:** N/A	Adusumilli et al.^168^
	LCAR-M23 cells	NCT04562298∗	I	2022-06-07 (terminated due to sponsors and collaborator decision)	15	Shanghai East Hospital	evaluation of safety, tolerability, pharmacokinetics, and efficacy of LCAR-M23 cells	EOC	**posology:** LD. Single-dose injection **safety:** N/A **efficacy:** N/A	–
	PD-1 KO anti-MSLN CAR-TILs secreting anti-PD-1/CTLA-4 scFvs	NCT04842812	I	estimated 2035-01-01	40	Second Affiliated Hospital of Guangzhou Medical University	evaluation of safety, tolerability, and efficacy of PD-1 KO anti-MSLN CAR-TILs secreting anti-PD-1/CTLA-4 scFvs	Solid	**posology:** No LD. 1 to 10 × 10^7^ cells/kg for each treatment, 3 or more cycles **safety:** N/A **efficacy:** N/A	–
	anti-MSLN CAR-T cells	NCT05531708∗	I	2021-04-02 (withdrawn)	0	Shanghai Pudong Hospital	evaluation of safety and efficacy of anti-MSLN CAR-T cells	Solid	**posology:** LD. Single-dose injection i.v. **safety:** N/A **efficacy:** N/A	–
	anti-MSLN CAR-T cells (mRNA transfected CAR-T cells)	NCT04981691∗∗	I	estimated 2022-07-09 (unknown status)	12	Ruijin Hospital	evaluation of safety, pharmacokinetics, and efficacy of anti-MSLN CAR-T cells	Solid	**posology:** LD. First week, 1 to 3 × 10^9^ cells i.v. divided into 3 injections. Second week, 1 to 3 × 10^9^ cells i.v. for each injection (3 injections) **safety:** N/A **efficacy:** N/A	–
CCAR-T cells	FH-TCR-Tᴍsʟɴ cells	NCT04809766	I	2025-01-15	9	Fred Hutchinson Cancer Center	evaluation of safety, pharmacokinetics, and efficacy of FH-TCR-Tᴍsʟɴ	PDAC	**Data cutoff date: 2023-07-01** **posology:** LD. 3 injections of 1 × 10^9^ or 3.3 × 10^9^ cells Q3W **safety (*n* = 6):** no DLTs occurred. 17% of patients experienced SAEs **efficacy:** N/A	Chiorean et al.^170^
	RD133 cells	NCT05166070	I	estimated 2037-01-01	24	The First Affiliated Hospital with Nanjing Medical University	evaluation of safety, tolerability, and efficacy of RD133 cells	solid	**posology:** LD. Single-dose injection of 1–6 × 10^6^ cells/kg i.v. **safety:** N/A **efficacy:** N/A	–
	LD013 cells	NCT05372692	N/A	2023-02-01	3	Weijia Fang, MD	evaluation of safety of LD013 cells	ovarian	**posology:** no LD **safety:** N/A **efficacy:** N/A	–
	TC-510 cells (lentiviral transduced anti-MSLN sdAb (MH1)/CD3ζ TCR-T cells with PD-1:CD28 switch receptor)	NCT05451849	I/II	estimated 2028-10-30	6	TCR2 Therapeutics	evaluation of safety and efficacy of TC-510	solid	**posology:** LD **safety:** N/A **efficacy:** N/A	McCarthy et al.^171^
	BZE2209 cells (anti-MSLN CAR-T cells secreting anti-PD-1/CTLA-4 sdAbs)	NCT06248697	I	estimated 2026-12-31	16	Shanghai Cell Therapy Group	evaluation of safety and tolerability of BZE2209 cells	solid	**posology:** no LD. i.v. **safety:** N/A **efficacy:** N/A	–
		NCT05783089	I	estimated 2027-04-01	87	Cancer Institute and Hospital, Chinese Academy of Medical Sciences	evaluation of safety, tolerability, and efficacy of anti-MSLN CAR-T cells	Solid	**posology:** LD. Single-dose injection of 0.1–2 × 10^6^ cells/kg i.v. or local injection, w/ or w/o checkpoint inhibitors **safety:** N/A **efficacy:** N/A	–
		NCT05848999∗∗	I	estimated 2025-07-01 (unknown status)	87	Zhejiang University	evaluation of safety, tolerability, and efficacy of UCLM802	Solid	**posology:** LD. Single-dose injection of 0.1–2 × 10^6^ cells/kg i.v. **safety:** N/A **efficacy:** N/A	–
		NCT05775666	I	estimated 2025-01-01	87	Peking University	evaluation of safety, tolerability, and efficacy of UCLM802 cells	Solid	**posology:** LD **safety:** N/A **efficacy:** N/A	–
	anti-MSLN CAR-T cells secreting IL-21 and anti-PD-1 scFv	NCT05779917	I	estimated 2036-03-10	30	Second Affiliated Hospital of Guangzhou Medical University	evaluation of safety, tolerability, and efficacy of anti-MSLN CAR-T cells secreting IL-21 and anti-PD-1 scFv	pancreatic	**posology:** no LD **safety:** N/A **efficacy:** N/A	–
CAR-T cells	SynKIR-110 cells (anti-MSLN KIR-CAR-T cells)	NCT05568680	I	estimated 2027-12-01	42	Verismo Therapeutics	evaluation of feasibility, safety, tolerability, and efficacy of SynKIR-110 cells	ovarian, CHO, and mesothelioma	**posology:** no LD. Single-dose injection i.v. **safety:** N/A **efficacy:** N/A	–
		NCT06701201	N/A	estimated 2042-02-01	60	Verismo Therapeutics	evaluation of long-term safety and efficacy of SynKIR-110 cells	N/A	**posology:** N/A **safety:** N/A **efficacy:** N/A	–
		NCT06377202∗	N/A	2023-11-15 (withdrawn and replaced by NCT06701201)	0	Verismo Therapeutics	evaluation of long-term safety of SynKIR-110 cells	Solid	**posology:** no LD. Single-dose injection **safety:** N/A **efficacy:** N/A	–
	BZT2312 cells (anti-MSLN CAR-T cells secreting anti-PD-1 sdAb)	NCT06249256	I	estimated 2026-12-31	12	Shanghai Cell Therapy Group	evaluation of safety, tolerability, and efficacy of BZT2312 cells	Solid	**posology:** LD. Single-dose injection of 5 × 10^5^ to 5 × 10^6^ cells/kg i.v. **safety:** N/A **efficacy:** N/A	–
	anti-MSLN TCR-like CAR-T cells (retroviral transduced CAR-T cells)	NCT05963100	I/II	estimated 2026-05-16	10	Zhongda Hospital	evaluation of safety and efficacy of anti-MSLN TCR-like CAR-T cells	Ovarian	**safety:** No LD. 1 × 10^6^ to 2 × 10^7^ cells/kg **safety:** N/A **efficacy:** N/A	–
	anti-MSLN CAR-T cells	NCT06054308∗	N/A	2024-02-02 (withdrawn)	0	Chinese University of Hong Kong	evaluation of feasibility of endoscopic ultrasound-guided injection of anti-MSLN CAR-T cells	PDAC	**posology:** no LD. Endoscopic ultrasound-guided injection **safety:** N/A **efficacy:** N/A	–
	UCMYM802 cells (mRNA transfected anti-MSLN CAR-T cells)	NCT06256055	I	estimated 2025-04-01	24	UTC Therapeutics	evaluation of safety, tolerability, pharmacokinetics, and efficacy of UCMYM802 cells	solid	**posology:** 1 × 10^8^ to 2 × 10^9^ cells i.v. QW (4 injections) **safety:** N/A **efficacy:** N/A	–
	BZE2203 cells (anti-MSLN CAR-T cells secreting anti-PD-1 sdAb)	NCT06327997	I	estimated 2027-12-31	20	Shanghai Cell Therapy Group	evaluation of safety, tolerability, pharmacokinetics, and efficacy of BZE2203 cells	Solid	**posology:** no LD. Single-dose injection of 5 × 10^5^ to 5 × 10^6^ cells/kg i.v. **safety:** N/A **efficacy:** N/A	–
	A2B694 cells (logic-gated Tmod™ CAR-T cells with anti-MSLN activating receptor and HLA-A∗02 blocking receptor)	NCT06051695	I/II	estimated 2029-06-01	230	A2 Biotherapeutics	evaluation of safety, tolerability, and efficacy of A2B694 cells	solid	**posology:** LD. Single-dose injection i.v. **safety:** N/A **efficacy:** N/A	Punekar et al.^207^
	CHT102 cells (anti-MSLN UCAR-T cells)	NCT06760364	I	estimated 2039-12-24	21	Tianjin Medical University Cancer Institute and Hospital	evaluation of safety, pharmacokinetics, and efficacy of CHT102 cells	Pancreatic	**posology:** No LD. Arterial injection. **safety:** N/A **efficacy:** N/A	–
		NCT06717022	N/A	estimated 2039-05-01	24	Tianjin Medical University Cancer Institute and Hospital	evaluation of safety and efficacy of CHT102 cells	N/A	**posology:** no LD. 2.5 × 10^6^ to 3 × 10^7^ cells/kg i.v. on days 1, 5, and 9 **safety:** N/A **efficacy:** N/A	–
CAR-T cells	MSLN-CAR-γδT cells	NCT06196294	I	estimated 2036-12-30	30	Second Affiliated Hospital of Guangzhou Medical University	evaluation of safety, tolerability, and efficacy of MSLN-CAR-γδT cells	pancreatic, lung, liver, and mesothelioma	**posology:** no LD **safety:** N/A **efficacy:** N/A	–
CAR-PBMCs	MCY-M11 cells (mRNA transfected CAR-PBMCs)	NCT03608618∗	I	2021-08-24 (terminated due to shift of sponsor’s focus)	14	MaxCyte	evaluation of feasibility, safety, and tolerability of MCY-M11 cells administered intraperitoneally	solid	**Data cutoff date: 2020-01-01** **posology:** no LD. Injections of 1 × 10^7^ to 5 × 10^8^ cells i.p. QW for 3 weeks **safety (*n* = 11):** no TRAEs reported. No DLTs reported **efficacy at 5 × 10**^**7**^**(*n* = 3):** PFS: 4 months. OR: CR 0%, PR 0%, SD 100%, and PD 0%.	Annunziata et al.^172^
CAR-NK cells	anti-MSLN CAR-NK cells	NCT03692637∗∗	I	estimated 2021-11-01 (unknown status)	30	Allife Medical Science and Technology	evaluation of safety, tolerability, and efficacy of anti-MSLN CAR-NK cells	EOC	**posology:** no LD. Single-dose injection of 5 × 10^5^ to 3 × 10^6^ cells/kg i.v. **safety:** N/A **efficacy:** N/A	–
	anti-MSLN CAR-NK cells; anti-MSLN CAR-NK cells secreting IL-7/CCL19; anti-MSLN CAR-NK secreting anti- PD-1/PD-L1/CTLA-4 scFvs	NCT05410717	I	estimated 2036-05-31	200	Second Affiliated Hospital of Guangzhou Medical University	evaluation of safety and efficacy of anti-MSLN CAR-NK cells; anti-MSLN CAR-NK cells secreting IL-7/CCL19; anti-MSLN CAR-NK secreting anti- PD-1/PD-L1/CTLA-4 scFvs alone and in combination with cannabidiol or nicotinamide adenine dinucleotide	ovarian, testis, and endometrial	**posology:** no LD **safety:** N/A **efficacy:** N/A	Li et al.^208^
CAR-macrophages	TAK-103 cells	NCT05164666	I	estimated 2027-10-31	2	Takeda	evaluation of safety, pharmacokinetics, and efficacy of TAK-103 cells	solid	**posology:** no LD. Single-dose injection of 1 × 10^6^ to 5 × 10^8^ cells i.v. **safety:** N/A **efficacy:** N/A	–
	SY001 cells(adenoviral transduced anti-MSLN scFv/4-1BB/CD3ζ CAR-macrophages)	NCT06562647	N/A	estimated 2025-04-01	2	CellOrigin Biotech	evaluation of safety, tolerability, pharmacokinetics, and efficacy of SY001 cells in combination with tislelizumab	ovarian	**posology:** no LD. SY001 cells injections i.v. on days 1, 3, and/or 5 + tislelizumab i.v. on day 1 **safety (*n* = 2):** 100% of patients experienced transient cytokine fluctuations and reductions in lymphocytes or neutrophils post-treatment but no other grade ≥3 AEs or CRS were observed **efficacy (*n* = 2):** OR: SD 100% (28-day follow-up period)	Li et al.^173^

Discontinued clinical trials are shown with the symbol ∗. Clinical trials with an unknown status are shown with the symbol ∗∗. AE, adverse event; CHO, cholangiocarcinoma; CR, complete response; CRS, cytokine release syndrome; DLT, dose-limiting toxicity; EOC, epithelial ovarian cancer; i.p., intrapleural; itu., intratumoral; i.v., intravenous; LD, lymphodepletion; MPM, malignant pleural mesothelioma; NE, not evaluated; NSCLC, non-small-cell lung cancer; OR, overall response; OS, overall survival; PD, progressive disease; PDAC, pancreatic ductal adenocarcinoma; PFS, progression-free survival; PR, partial response; Q3W, once every 3 weeks; QW, once weekly; RP2D, recommended phase 2 dose; SAE, serious adverse event; SD, stable disease; TNBC, triple-negative breast cancer; TRAE, treatment-related adverse event.

Different strategies have been developed to improve MSLN CAR-T cells efficiency and address the immunosuppressive TME and CAR-T cell exhaustion: (1) CAR-T cells secreting ICI, cytokine, or chemokine; (2) multispecific CAR-T cells; (3) logic-gated CAR-T cells; (4) allogenic CAR-T cells; and (5) PD-1 KO CAR-T cells. Different routes of administration are also being evaluated to improve tumor targeting and reduce systemic toxicity, notably regional delivery such as intrapleural or intraperitoneal administration. In some cases, CAR-T cells have undergone modifications during their development. For instance, M28z cells have been updated to M28z1XXPD1DNR, featuring a modified CD3ζ and a PD-1 dominant negative receptor. Similarly, αPD-1-mesoCAR-T cells, initially developed to secrete anti-PD-1 antibody, have been re-engineered to secrete anti-PD-1 sdAb.^167^^,^^168^^,^^169^

Anti-mesothelin CAR-T trials are mainly in the early stages (phase I/II), to assess safety, tolerability, and early signs of efficacy. Data on the design and available results are limited. However, the results published to date show acceptable tolerability and clinical responses in certain patients, notably in malignant pleural mesothelioma using regional intrapleural delivery, particularly when combined with pembrolizumab.^167^

Beyond the traditional CAR-T cell approach, alternative strategies for engineering T cells have also been explored. These include (1) T-cell-receptor-engineered T cells (TCR-T cells), in which patient TCR is genetically modified to recognize a mesothelin-specific peptide presented by a specific human leukocyte antigen (HLA) molecule, typically HLA-A∗02:01, as exemplified by the FH-TCR-T_MSLN_ cells developed at the Fred Hutchinson Cancer Center, and (2) T cell receptor fusion construct (TRuC) T cells, which involve the fusion of an anti-mesothelin sdAb directly to the CD3ε subunit of the native CD3/TCR complex.^170^^,^^171^ This platform has been tested both without (NCT03907852) and with (NCT05451849) the co-expression of a chimeric switch receptor designed to convert inhibitory signals from the TME, such as PD-L1, into stimulatory signals for the T cell.^171^

To date, a single clinical study has been conducted on CAR-γδT cells, and no data are yet available.

#### Other immune cell types: CAR-PBMCs, CAR-NK cells, and CAR-macrophages

Since 2018, there has been an expansion of research interest on immune cell populations other than T cells, which has led to the development of novel cell therapy approaches (Table 3).

A single clinical study has been conducted on CAR-PBMCs targeting MSLN. However, this study was prematurely stopped due to a change in the sponsor’s strategic priorities. Despite an acceptable tolerability, the available data fail to provide compelling evidence of efficacy.^172^

Two exploratory clinical studies have been conducted with autologous CAR-NK cells for evaluation of safety issues and preliminary efficacy mainly in gynecological cancers. No results have yet been published.

In addition, two clinical studies have been conducted on CAR-macrophages, with results only available for SY001 cells in combination with tislelizumab (anti-PD-1). The study’s findings revealed that 100% of patients experienced grade ≥3 adverse events (AEs), and no responses to the treatment were observed.^173^

## Conclusion

Mesothelin has been established as a compelling target due to its high expression in several aggressive tumors and limited expression in healthy tissues. Over recent years, a broad range of mesothelin-targeted therapies have progressed into clinical trials, with satisfactory safety profiles and encouraging early signs of efficacy, notably in mesothelioma, with consistent objective radiologic responses following administration of anti-mesothelin CAR-T cells.^174^

However, despite substantial supporting evidence for MSLN-targeted therapies (Tables 1, 2, and 3), several factors may collectively contribute to their modest clinical outcomes.

The “antigen sink” phenomenon, driven by the presence of soluble mesothelin in both tumor tissue and circulation, is frequently cited as a contributing factor to the heterogeneous outcomes observed across clinical trials. However, its impact remains poorly documented due to the inconsistent measurement, reporting, and consideration of circulating mesothelin levels in clinical studies. To mitigate the decoy effect of soluble mesothelin, strategies targeting membrane proximal epitopes have been developed, including the engineering of bispecific antibodies and CAR-T cells designed to preferentially engage membrane-bound targets despite the presence of soluble antigen.^175^

Patient selection in clinical trials should take into account prior exposure to chemotherapy. Several studies have shown that chemotherapy can stimulate tumor cells to activate proteases such as ADAM17, which is implicated in mesothelin shedding.^176^^,^^177^ This increase in protease activity may raise sMSLN levels. Therefore, it would be valuable to stratify patients based on their chemotherapy history and measure baseline sMSLN. Such stratification could help interpret therapeutic responses and guide dose-exposure analyses more accurately.

It should be noted that the occurrence of soluble forms of membrane receptors in patient serum is not exclusive to mesothelin. Soluble form of many TAAs have been detected, some of them (EGFR, CEA, and HER2) at concentrations comparable to those of mesothelin.^178^^,^^179^^,^^180^ Immune checkpoint receptors, including PD-1, PD-L1, and CTLA-4, are also present as circulating molecules.^181^^,^^182^ These soluble factors can either hinder or enhance antitumor therapy efficacy depending on their concentrations, context, and interactions within the TME.

Although mesothelin targeting is primarily considered in its membrane-associated form, the soluble fraction may also represent a therapeutically relevant target. From a therapeutic standpoint, engaging soluble MSLN could facilitate passive diffusion within the tumor and help mitigate classical limitations such as the binding site barrier, thereby improving tissue penetration.^183^^,^^184^ In addition, certain therapeutic modalities, particularly nuclear medicine approaches such as beta-emitting radiotherapy, do not necessarily require direct membrane engagement to achieve cytotoxic effects, which further supports the potential interest of targeting non-membrane-associated MSLN.^185^

Another important but often insufficiently documented aspect in clinical trial reports is the MUC16 status of enrolled patients. Beyond its role in contributing to local immunosuppression and affecting therapeutic efficacy, MUC16 may modulate the retention of circulating mesothelin in tumors by facilitating mesothelin rebinding to the surface of MUC16-expressing tumor cells, resulting in localized mesothelin enrichment.^34^^,^^186^ However, both membrane-bound and soluble forms of MUC16/CA125 can interfere with the antibody recognition of mesothelin, particularly for antibodies targeting epitopes overlapping with the MUC16-binding site, thereby impacting patient stratification.

Additional factors may also be considered, including the heterogeneity of MSLN expression, which likely contributes to therapeutic resistance, as well as the association of high MSLN expression with a cold and immunosuppressive TME, characterized by limited infiltration of cytotoxic T cells and upregulation of immune checkpoint molecules such as PD-L1.

In parallel, several therapeutic failures have stemmed from molecule-intrinsic limitations rather than from the mesothelin target itself. Immunotoxins were restricted by immunogenicity and systemic toxicity,^129^^,^^133^ while amatuximab, though well tolerated, exhibited reduced effector functions due to MUC16-MSLN and MUC16-Fab interactions.^154^^,^^155^ In addition, the stroma of solid tumors may limit drug penetration and therapeutic efficacy. Conversely, early-phase clinical trials of mesothelin-directed CAR-T cells have reported encouraging outcomes though larger studies remain necessary to confirm their long-term safety and effectiveness.

Interestingly, these limitations are largely representative of the obstacles faced by tumor-antigen-targeted therapies in the context of solid tumors.

A better understanding of the biological and clinical implications of mesothelin, combined with the systematic integration of circulating mesothelin measurement and MUC16 status in clinical trials, could enhance the interpretation of results and enable more appropriate patient selection.

Several emerging research directions are being pursued to develop novel mesothelin-targeting therapies addressing current limitations and aiming to enhance efficacy, safety, and durability.

Innovative approaches such as conditional mesothelin binding via dual or multi-targeting and affinity tuning are currently evaluated.^187^^,^^188^^,^^189^^,^^190^ Moreover, although amatuximab alone is not effective, the “theranostic strategy,” combining ^89^Zr- and ^177^Lu-labeled amatuximab, has shown promise with *in vivo*-specific tumor accumulation in PANC-1 MSLN^+^ models and therapeutic efficacy with minimal side effects.^191^

In summary, mesothelin remains an attractive therapeutic target, offering considerable hope for the treatment of aggressive cancers. Nonetheless, it continues to hold many unresolved questions, making it a particularly stimulating field for future research.

## Acknowledgments

R.B. was supported by 10.13039/100007586Aix Marseille University. P.C. and B.K. were supported by 10.13039/501100004794CNRS. This work was supported by institutional grants from 10.13039/501100004794CNRS and 10.13039/501100001677Inserm.

## Author contributions

R.B., conceptualization, literature search and data curation, formal analysis, visualization, writing—original draft, and writing—review & editing. P.C., validation. B.K., conceptualization, formal analysis, writing—review & editing, supervision, and validation. All authors reviewed and approved the final version of the manuscript.

## Declaration of interests

The authors have declared that no competing interest exists.
